# Modelling the impacts of climate change on riverine thermal regimes in western Canada’s largest Pacific watershed

**DOI:** 10.1038/s41598-019-47804-2

**Published:** 2019-08-06

**Authors:** Siraj Ul Islam, Rachel W. Hay, Stephen J. Déry, Barry P. Booth

**Affiliations:** Environmental Science and Engineering Program, University of Northern British Columbia, Prince George, British Columbia Canada

**Keywords:** Hydrology, Hydrology

## Abstract

Quantification of climate change impacts on the thermal regimes of rivers in British Columbia (BC) is crucial given their importance to aquatic ecosystems. Using the Air2Stream model, we investigate the impact of both air temperature and streamflow changes on river water temperatures from 1950 to 2015 across BC’s 234,000 km^2^ Fraser River Basin (FRB). Model results show the FRB’s summer water temperatures rose by nearly 1.0 °C during 1950–2015 with 0.47 °C spread across 17 river sites. For most of these sites, such increases in average summer water temperature have doubled the number of days exceeding 20 °C, the water temperature that, if exceeded, potentially increases the physiological stress of salmon during migration. Furthermore, river sites, especially those in the upper and middle FRB, show significant associations between Pacific Ocean teleconnections and regional water temperatures. A multivariate linear regression analysis reveals that air temperature primarily controls simulated water temperatures in the FRB by capturing ~80% of its explained variance with secondary impacts through river discharge. Given such increases in river water temperature, salmon returning to spawn in the Fraser River and its tributaries are facing continued and increasing physical challenges now and potentially into the future.

## Introduction

**Figure 1:**
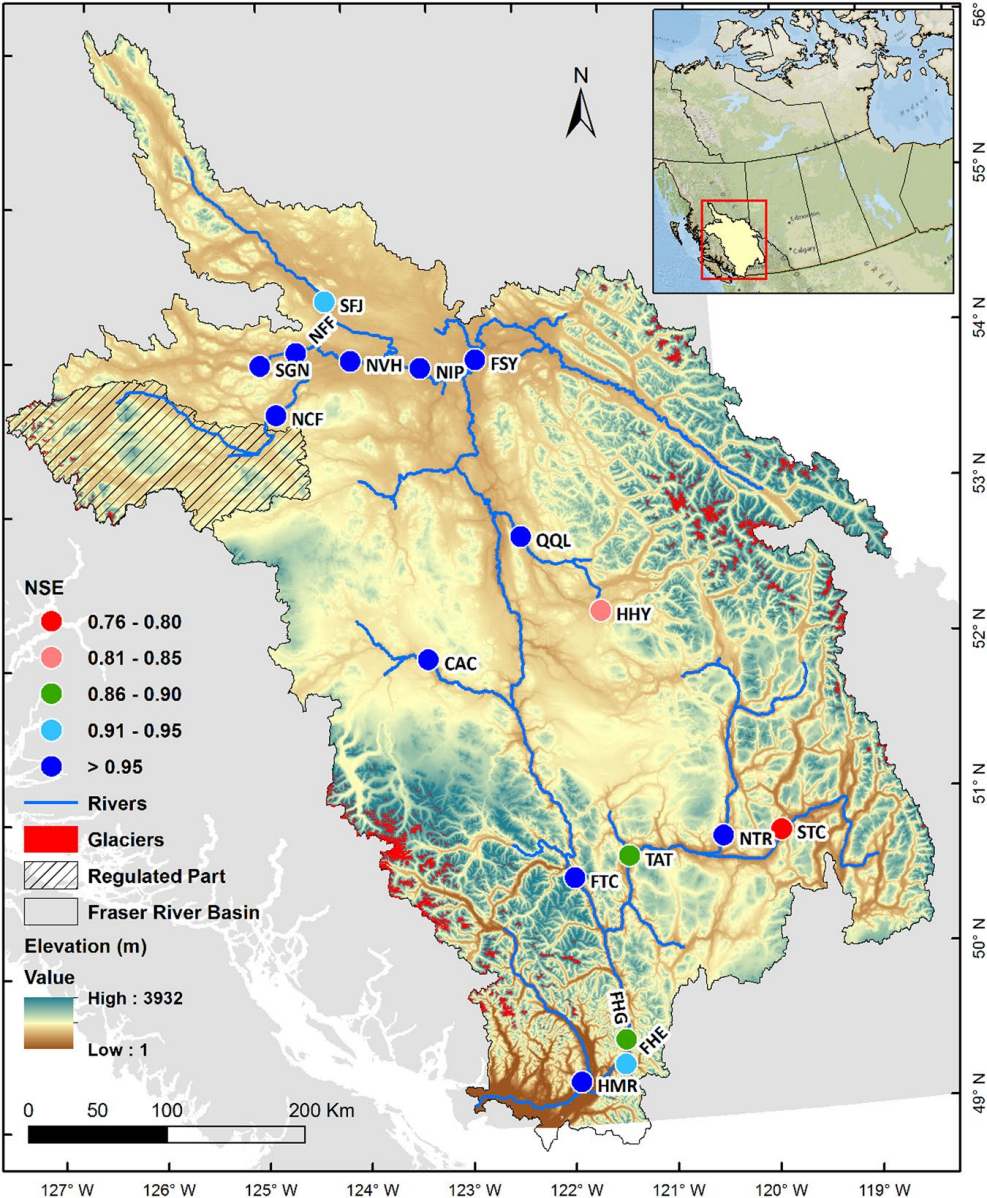
Map of the FRB in BC showing the 17 selected river sites (See Table 1 for the abbreviations). These sites were used to apply quality control and analysis on available observed water temperature time series and implementation of the Air2Stream model. Colours represent the Nash-Sutcliffe Efficiency (NSE) values revealing the performance of the model simulations during the calibration time period as reported in Supplementary Table S3. Map generated using ESRI ArcMap 10.5.1 (http://desktop.arcgis.com/en/arcmap/).

The FRB, western Canada’s largest Pacific River watershed, spans one-quarter of BC from its headwaters in the Rocky Mountains to its outlet to the Pacific Ocean at Vancouver^1^ (Fig. 1). Collecting water from its vast network of tributaries, it accumulates Canada’s third-highest mean annual flow (3,972 m^3^ s^−1^), which is largely snowmelt-driven^1^. The Fraser River is a critically important waterway for BC’s communities, ecosystems, and economy. It remains one of the most important wild salmonid-producing rivers globally and supports runs of five species of Pacific salmon: Chinook, chum, coho, pink and sockeye^2,3^. Among these, sockeye is the most important both culturally and economically^4,5^. Salmon spawn as far inland as 1500 km from the mouth of the Fraser and their successful return to natal spawning areas depends on seasonal variability of flows and water temperature^6,7^. As a result, natural or human-induced changes in the flow and/or thermal characteristics of the FRB can induce considerable impacts on these species.

Climate change impacts have been observed across the FRB where observed air temperatures have risen 1.4 °C (1949–2006)^8^ and up to 2.3 °C (1970–2009) in parts of the Nechako sub-watershed^9,10^. In response to such warming in the latter half of the 20^th^ century and early 21^st^ century, river flows throughout the FRB show increased variability due to changes in precipitation patterns and phase^8^, glacier retreat, along with climatic phenomena such as El Niño-Southern Oscillation (ENSO) and Pacific Decadal Oscillation (PDO)^11^. Warming air temperatures combined with changing flow characteristics have major impacts on river water temperature^12^ and subsequently on aquatic species^13^ that are sensitive to thermal changes. While various factors control river water temperature^12–14^, the mean daily air temperature remains a key driver in modulating water temperatures, and conditions that are critical for successful salmonid survival in freshwater. Mean daily air temperatures and water temperatures are traditionally related as a joint system that exchanges net heat under an equilibrium temperature assumption^15,16^. While there is considerable research interest in both observed climate change and impacts on salmon species in the FRB, further work is needed to provide a comprehensive understanding of the large spatial and temporal climatic patterns that exert an influence on the FRB and how these relate to river water temperature.

Several studies have investigated the changes in water temperatures by examining either the limited historical water temperature records^17,18^ or water temperature reconstructions^19–21^ of the Fraser River and its tributaries. The reconstruction efforts include correlation analysis of summer water temperature and flows in relation to Early Stuart, Early Summer and Summer sockeye runs in the FRB^19^, regression and logistic-based modelling in the Chilko-Chilcotin^20^ system, linear regression and neural network techniques in the lower Fraser^21^ and water temperature reconstructions for the lower Fraser sockeye runs using long-term measurement sites and nearby water temperature datasets^5^. To provide meaningful insights into water temperature changes in this region, more advanced modelling applications are needed, especially where observational data are limited on geographic and temporal scales. For example, there is only one site across the entire FRB with observed daily water temperature records for the complete study period of 1950–2015. The lack of comprehensive water temperature data limits our understanding of long-term river thermal changes, their predictability and the potential impact on species such as salmon. Insufficient data records also limit the analysis of Pacific Ocean climate variability that potentially modulates water temperatures at regional scales.

In this study, a state-of-the-art hybrid river water temperature model (Air2Stream^22^) is used to simulate daily river water temperature as a function of gridded air temperature (extracted from spatially interpolated observations of minimum and maximum daily air temperatures) and observed discharge at 17 river sites within the FRB from 1950 to 2015. The Air2Stream model was previously used in several river basins globally^23–26^ including the Mackenzie and Yukon River basins in Canada with an ability to perform better than statistical regression models^27^. In this study, using simulated water temperatures, we estimate summer trends, timing of peak water temperatures and occurrence of river temperatures above critical thresholds (i.e. the temperature range where salmon become stressed thereby affecting migration success^28^) to document changes in river thermal characteristics in relation to their possible impacts on sockeye salmon. To our knowledge this is the first study of its kind to simulate water temperatures simultaneously in various salmonid rivers across the FRB using this approach. This study thus addresses three main research aims: (1) to implement and evaluate a hybrid water temperature model at 17 river sites in the FRB, (2) to develop a comprehensive water temperature dataset to analyze long-term spatio-temporal changes in river water temperatures on various timescales, and (3) to quantify changes in extreme water temperature that may impact salmon migration.

## Methods

Observed river water temperature data were acquired from a variety of sources including Triton Environmental Consultants, the Department of Fisheries and Oceans Canada, and the Water Survey of Canada. Supplementary Table S1 lists the observed water temperature databases along with data provider and location. All river sites (Fig. 1) had at least five years of observed daily river water temperature during summer excluding the HMR river site that had only four years of data. Locations for analyses were selected based on drainage area, data availability, and importance to sockeye salmon runs^4^. These sites encompassed a wide range of elevations, basin sizes, and climatic conditions across BC. We used observation-based daily gridded climate data (referred to as ANUSPLIN) interpolated using the method implemented by the Australian National University spline interpolation^29,30^ to extract air temperatures for each river site. This Canada-wide product was developed by Natural Resources Canada (NRCan) and contains gridded station-based data of daily minimum and maximum air temperature (°C) at ~10 km resolution. We averaged daily minimum and maximum air temperature to obtain the daily mean air temperature as commonly used in the literature^27^. Observed daily discharge data were acquired from Water Survey of Canada measurement locations. Details of the hydrometric gauges and time periods are provided in Supplementary Table S2.

Similar quality control methods were applied to time series of river discharge and water temperature data. The first step involved the removal of physically impossible outliers such as values <0 °C for river water temperature, followed by two gap-filling methods for missing data. Linear interpolation was used to fill gaps of six days or less. Decadal climatologies were calculated and the daily values from the climatologies were used for filling gaps of seven days or longer. Finally, datasets and climatologies were plotted to identify any anomalous data before and after gap-filling. For some sites, discharge time series from different sources were combined to fill extended gaps or, if no data were available, data from the nearest site were used (see Supplementary Information for further details).

### Air2Stream model and its implementation

To simulate daily water temperatures, we employed the Air2Stream model^22^, a state-of-the-art hybrid river water temperature model relying on heat budgets with a similar formulation and approach as a lake surface temperature model^31,32^. The model utilizes an unknown river reach of volume V and atmospheric heat exchanges by implicitly considering both surface and subsurface water fluxes^22^. The formulation of the Air2Stream model begins from the physical relationships that are simplified to form a single ordinary differential equation linearly dependent on air temperature, water temperature and discharge. It is therefore considered a physically-based tool utilizing the observed data to update its numerical integration^22^. The model is widely used with several recent studies comparing Air2Stream output with statistical models^24,27^, assessing regulation impacts on water temperature^25^ and exploring impacts of thermal and flow regimes changes on early life stages of salmonids^26^.

The Air2Stream model was integrated for its calibration and validation (Supplementary Tables S3 and S4) using daily gridded air temperature, water temperature and discharge data at 17 river sites within the FRB (Fig. 1, Table 1). At each site we ran the Air2Stream model with 3-, 5- or 8-parameters to select its optimal version for which results are reported herein. Model performance was evaluated by comparing simulated daily water temperature time series with corresponding observations using the Nash-Sutcliffe efficiency (NSE) coefficients, root mean square error (RMSE) and mean bias (BIAS) statistical metrics. We also assessed model performance by comparing the observed and simulated water temperature interannual variability and, where data allow, long-term trends. This step allowed verification of Air2Stream’s performance in reproducing water temperature fluctuations associated with interannual climate oscillations such as ENSO and PDO and long-term trends owing to climate change. Supplementary Fig. S1 describes the full experimental setup of the Air2Stream model implementation and application.

**Table 1:** River water temperature sites, names, coordinates, mean basin elevation and Air2Stream model parameters used for each site.

Site	Name	Latitude (°N)	Longitude (°W)	Mean Basin Elevation (m)	Air2Stream Number of Parameters
SFJ	Stuart River at Fort St. James	54.41	124.27	1097	8
NFF	Nautley River at Fort Fraser	54.08	124.60	1070	8
NCF	Nechako River below Cheslatta Falls	53.68	124.83	1223	5
SGN	Stellako River at Glenannan	54.00	125.00	1088	8
NVH	Nechako River at Vanderhoof	54.02	124.00	1152	8
NIP	Nechako River at Isle Pierre	53.96	123.23	1141	8
FSY	Fraser River at Shelley	54.00	122.62	1413	8
QQL	Quesnel River at Quesnel	52.84	122.22	1391	3
HHY	Horsefly River at Horsefly	52.33	121.41	1462	5
CAC	Chilcotin River at Alexis Creek	52.07	123.26	1528	5
FTC	Fraser River at Texas Creek	50.61	121.85	1303	5
NTR	North Thompson River at Rayleigh	50.82	120.30	1489	8
STC	South Thompson River at Chase	50.83	119.70	1315	8
TAT	Thompson River at Ashcroft	50.73	121.28	1363	5
FHG	Fraser River at Hells Gate	49.54	121.43	1320	8
FHE	Fraser River at Hope	49.38	121.45	1320	8
HMR	Harrison River below Morris River	49.28	121.90	1410	5

Following Toffolon and Piccolroaz^22^, we used the particle swarm optimization (PSO) algorithm^33^ with 2000 model iterations and 1000 population particles for each river site. Based on sensitivity runs using different optimization methods available within the model configuration, we found that the PSO method improves model simulations compared to other optimization approaches. The PSO method is a widely used calibration method^34,35^ with a Monte Carlo-based optimization procedure in which a large number of parameter sets are sampled and evaluated. The model was run using the numerical procedure of the Runge–Kutta 4th order discretization at daily time steps. Note that the calibration time period is different for several river sites based on available observed water temperature records (Supplementary Table S3). The calibration period varies from a maximum of ten years to a minimum of five years for some river sites with exception of the HMR river site where only four years of water temperature observations were available. Since the model calibration was conducted by minimizing water temperature differences against the observations, the simulated water temperature is not independent of the observed water temperature during the calibration time period.

Once the model was calibrated and validated, we integrated the model from 1950–2015 to estimate long-term daily time series for each river site and to evaluate changes in the simulated water temperatures. These simulations are referred as “hindcast simulations” in the text to differentiate them from the calibration and validation experiments reported in Supplementary Tables S3 and S4.

The Air2Stream model was driven using ANUSPLIN gridded air temperature forcing for all river sites. The ANUSPLIN gridded data are consistent and represent mean elevations at a ~10 km (area ~ 100 km^2^) grid scale. Overall, the main advantage of using ANUSPLIN is data homogeneity and lack of gaps, which are commonly reported as major issues with station-based data. We therefore extracted mean air temperature data from gridded observations for all 17 sites rather than using available limited station-based data.

### Analyses and statistical tests

Simulated mean summer water temperatures were first quantified for all river sites for 1950–2015. Monotonic trends in simulated water temperatures were then computed using the non-parametric Mann-Kendall Test (MKT)^36,37^. The statistical significance of the trends was estimated using a two-tailed test with *p* < 0.05. We summarised results using the temporal mean and interannual variability (standard deviation) of simulations at each site along with the multi-site mean and inter-site standard deviation (spread) of summer water temperature. The standard deviation at each site was used to compute signal to noise ratios (SNRs), i.e. 66-year summer water temperature trend divided by its interannual standard deviation.

We computed composites of strong El-Niño (1957–58, 1965–66, 1972–73, 1982–83, 1987–88, 1991–92, 1997–98, 2015–16) and strong La-Niña (1973–74, 1975–76, 1988–89, 1998–99, 1999–2000, 2007–08, 2010–11, 2011–12) years to assess the corresponding anomalies in water temperatures. Strong El-Niño and La-Niña years, based on the 3-month running mean sea surface temperature (SST) anomaly for the Niño 3.4 region (5°N-5°S, 170°W-120°W) in the tropical Pacific Ocean, were identified from National Oceanic and Atmospheric Administration (NOAA)^38^. The t-test statistic was used to compute the significance of the composite differences. Here it was assumed that the underlying process behaved as an independent and identically distributed random variable. We also computed differences between the strongest El-Niño (1997–98) and La-Niña (1973–74) events for each site to quantify corresponding anomalies in the water temperatures.

For the PDO association with water temperatures, we estimated simulated water temperature differences between the positive (1977–2002) and negative (1955–1976) phases of the PDO^38^. The t-test statistic was used for differences in conditions between positive and negative PDO phases.

Two 20-year time periods, namely 1956–1975 (referred hereafter as the 1960s) and 1996–2015 (referred hereafter as the 2000s) revealed climate change impacts on simulated water temperatures from the hindcast simulations. We used the frequency of exceeding different critical temperatures related to Pacific salmon physiology^19,39^. We considered days when simulated river water temperature exceeded 18 °C and 20 °C critical temperatures (T_c_) for different salmon species^40,41^. To account for climate change impacts on T_c_, we calculated the number of days greater or equal to T_c_ = 18 °C (T_c18_) and T_c_ = 20 °C (T_c20_) for the 1960s and 2000s in addition to the overall study period. This analysis also considered the impact of the Summer Temperature Management Project (STMP) in the Nechako River (NVH) by comparing results prior to and after its implementation in 1981. The STMP regulates water temperatures through additional releases of flows from the Nechako reservoir via the Skins Lake Spillway into the Nechako River for the benefit of salmon migration^42^.

Frequency distributions of water temperature for summer days utilized 50 equal bins with 0.5 °C spacing for each site in the 1960s and 2000s. To obtain a regional perspective of changes in distribution shape, simulated summer water temperature frequency distribution for seven, seven, and three sites were averaged for the upper (SFJ, NFF, NCF, SGN, NVH, NIP, FSY), middle (QQL, HHY, CAC, FTC, NTR, STC, TAT) and lower (FHG, FHE, HMR) FRB, respectively. The statistical significance of differences in frequency distributions was computed using a Kolmogorov‐Smirnov two‐sample test with *p* < 0.05.

### Multivariate linear regression analysis

To better understand the contributions of air temperature and river discharge to water temperatures, we used a multivariate linear regression (MLR) analysis, following a similar approach by Islam *et al*.^43^. We decomposed the summer water temperature *WT* (°C) into separate contributions from mean air temperature *T* (°C), discharge *Q* (m^3^ s^−1^) and residual *E* on mean summer and monthly bases, as follows:

1 $$W{T}_{n}={b}_{1}{T}_{n}+{b}_{2}{Q}_{n}+E,$$

where *b*_1_ and *b*_2_ are partial regression coefficients corresponding to the mean air temperature and discharge, respectively, and where subscript *n* = 1950, …, 2015 represents the year. The MLR was fitted for each site for mean summer and individual months independently using standardised and detrended monthly anomalies from 1950–2015. We standardized the time series to get zero mean and unit standard deviation. This was estimated by removing the mean and dividing the data by their standard deviation. The MLR explained variance R^2^ and standardized partial regression coefficients were computed along with their significance using t-test statistics. We only considered the lag-0 correlation between the driving and response variables in the MLR analysis considering that monthly time resolution should have encompassed any lags.

## Results

### Model performance

NSE, RMSE and BIAS scores provide measures of model performance in simulating daily river water temperature using the Air2Stream model^22^ across the FRB. Model results show that for all 17 study sites, NSE values range from 0.76 to 0.97 for the calibration period (average 0.91 ± 0.06), indicating high reliability of model simulations (Fig. 1). While the mean daily BIAS is small (0.07 °C, range −0.06 °C to 0.28 °C), daily RMSE scores average 1.26 °C and range between 0.85 °C to 2.04 °C across the FRB during the calibration period (Supplementary Table S3). Higher RMSE scores are mainly due to higher day-to-day variability in the observed data, which remains challenging for the Air2Stream model to simulate accurately. This may be due to the gap-filling and spatial smoothing procedures applied to generate continuous and homogenous daily air temperature in the ANUSPLIN dataset that may have suppressed rapid air temperature fluctuations. The resulting Air2Stream simulations therefore show dampened daily variability in simulated water temperatures when compared to observations. For the summer season on an interannual time scale, however, the model performs much better, particularly with respect to RMSE. During the calibration period summer RMSE values average 0.66 °C and range between 0.32 °C to 1.14 °C.

For the Air2Stream model validation, similar metrics cannot be computed for all sites due to limited water temperature observations. For example, the HMR observed water temperature data were available only for 2009–2015 and used to calibrate the Air2Stream model for this site; there were no data beyond this period for model validation. For river sites with extended observed water temperature records (e.g. NVH, FHG), we computed Air2Stream performance metrics for validation periods using at least five years of data (Supplementary Table S4). NSE scores for the validation period for these sites range from 0.80 to 0.97, with an average NSE score of 0.92 revealing high model reliability. Similar to the calibration period, daily RMSE scores exceed mean summer RMSE on an interannual time scale for all sites. During validation, daily RMSE averages 1.42 °C (ranging from 1.09 °C to 2.16 °C), which is slightly higher than the calibration mean RMSE score of 1.26 °C. Overall, the RMSE and BIAS scores are reasonably low enough (~≤2.0 °C) for all sites providing confidence that the Air2Stream model performs adequately during the calibration and validation time periods. Errors lie within the acceptable range of water temperature model reliability reported in other studies^21,22,27^. Observed and simulated water temperature climatologies during the calibration periods overlap each other for most of sites except FHE (Supplementary Fig. S2). Furthermore, the Air2Stream model shows robust performance in simulating the observed interannual variability in summer water temperature for most river sites (Supplementary Fig. S3). For example, the SFJ simulated summer mean river water temperatures vary coherently with observed water temperatures with ~2.0 °C mean difference in some years. At NFF and NVH where observed daily water temperature records exceed 30 years (1981–2015), the model reproduces well simulated short-term summer water temperature trends (Supplementary Fig. S3).

At FHG, the 66-year mean of simulated summer water temperature (16.10 °C) approaches the observed mean (15.97 °C) with a cross correlation between simulated and observed time series of 0.84. The model, however, underestimates the FHG trend magnitude compared to observations (0.98 °C (66 yr)^−1^ vs. 1.57 °C (66 yr)^−1^, respectively). Furthermore, the interannual variability of simulated summer water temperature is slightly lower than observed variability (Supplementary Fig. S4). The 66-year comparison between simulated and observed water temperatures is only possible for FHG considering the limited availability of long-term observations for all remaining sites. Comparison of simulated water temperature with gridded air temperature shows summer water temperatures nearly 2.0 °C to 4.0 °C warmer than air temperatures for most of river sites in the northern FRB. At the FSY, QQL, HHY, STC, TAT, FHG and HMR sites, however, mean summer water and air temperature magnitudes are almost similar, revealing these variables are strongly coupled at these sites.

### Summer water temperature trends and frequency distributions

**Figure 2:**
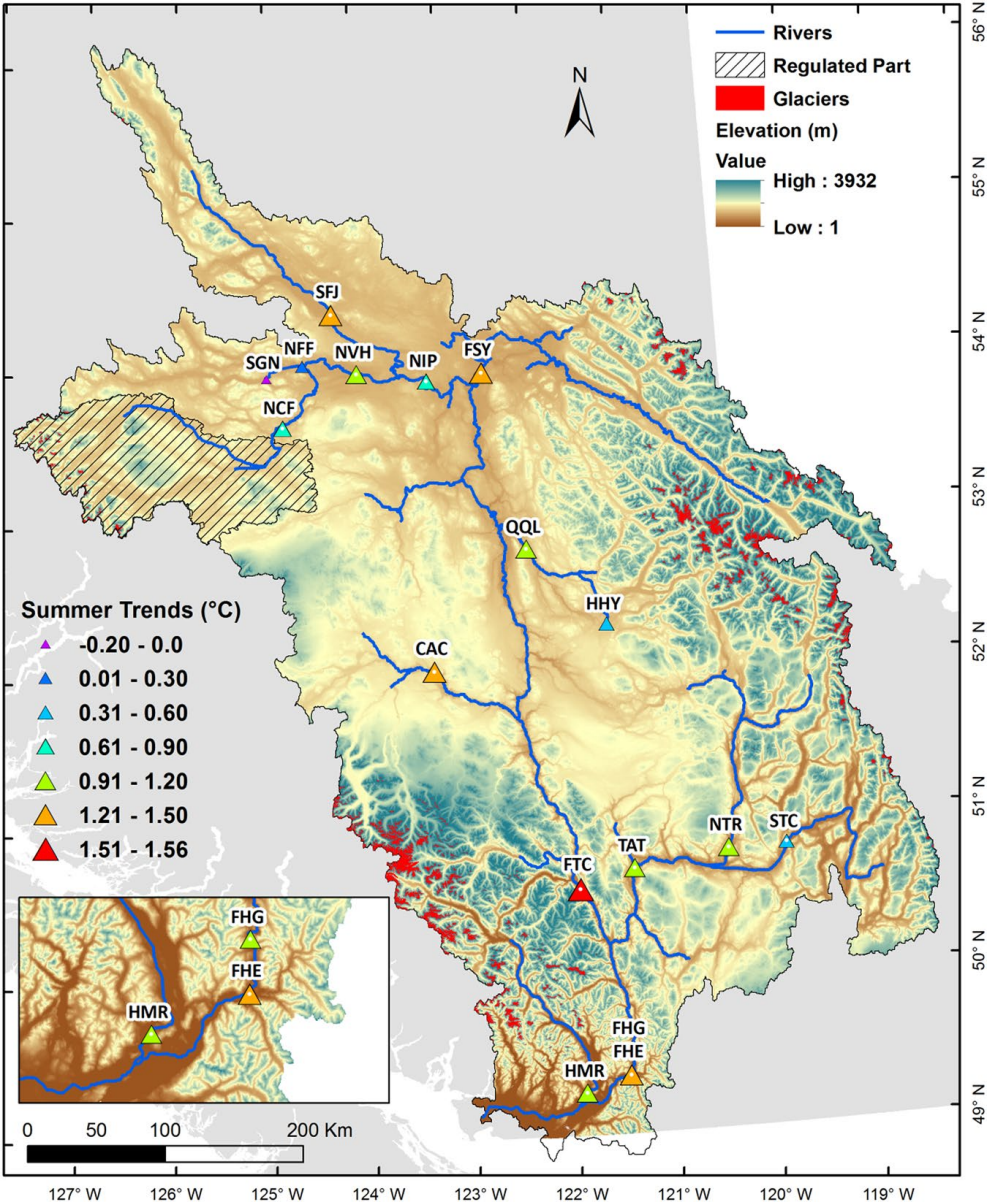
Simulated summer water temperature trends for 1950–2015 using the Mann-Kendall test (MKT). Triangle sizes represent the magnitude of the overall monotonic trend (°C (66 yr)^−1^). Triangles with white dots denote trends significant at *p* < 0.05 with *p*-values provided in Supplementary Table S5. Map generated using ESRI ArcMap 10.5.1 (http://desktop.arcgis.com/en/arcmap/).

All sites show significant (*p* < 0.05) warming trends in simulated summer river water temperatures (July to September) for 1950–2015 (Fig. 2) except NFF, SGN and HHY. The summer trend of 1.56 °C (66 yr)^−1^ is significantly (*p* < 0.05) highest at FTC (Supplementary Table S5). Most of the sites show significantly (*p* < 0.05) higher warming trends in August with the highest values at SFJ (1.68 °C (66 yr)^−1^), FSY (1.69 °C (66 yr)^−1^), CAC (1.75 °C (66 yr)^−1^) and FTC (1.80 °C (66 yr)^−1^) (Supplementary Table S5). The interannual variability of summer water temperature ranges between 0.37 °C to 0.88 °C across the FRB where the regulated sites in the Nechako River (NCF, NVH and NIP) lie in the bottom 50% of interannual variability (Supplementary Table S6). The signal to noise ratio exceeds unity for the sites with significant warming trends during summer suggesting this secular pattern dominates the interannual variation.

**Figure 3:**
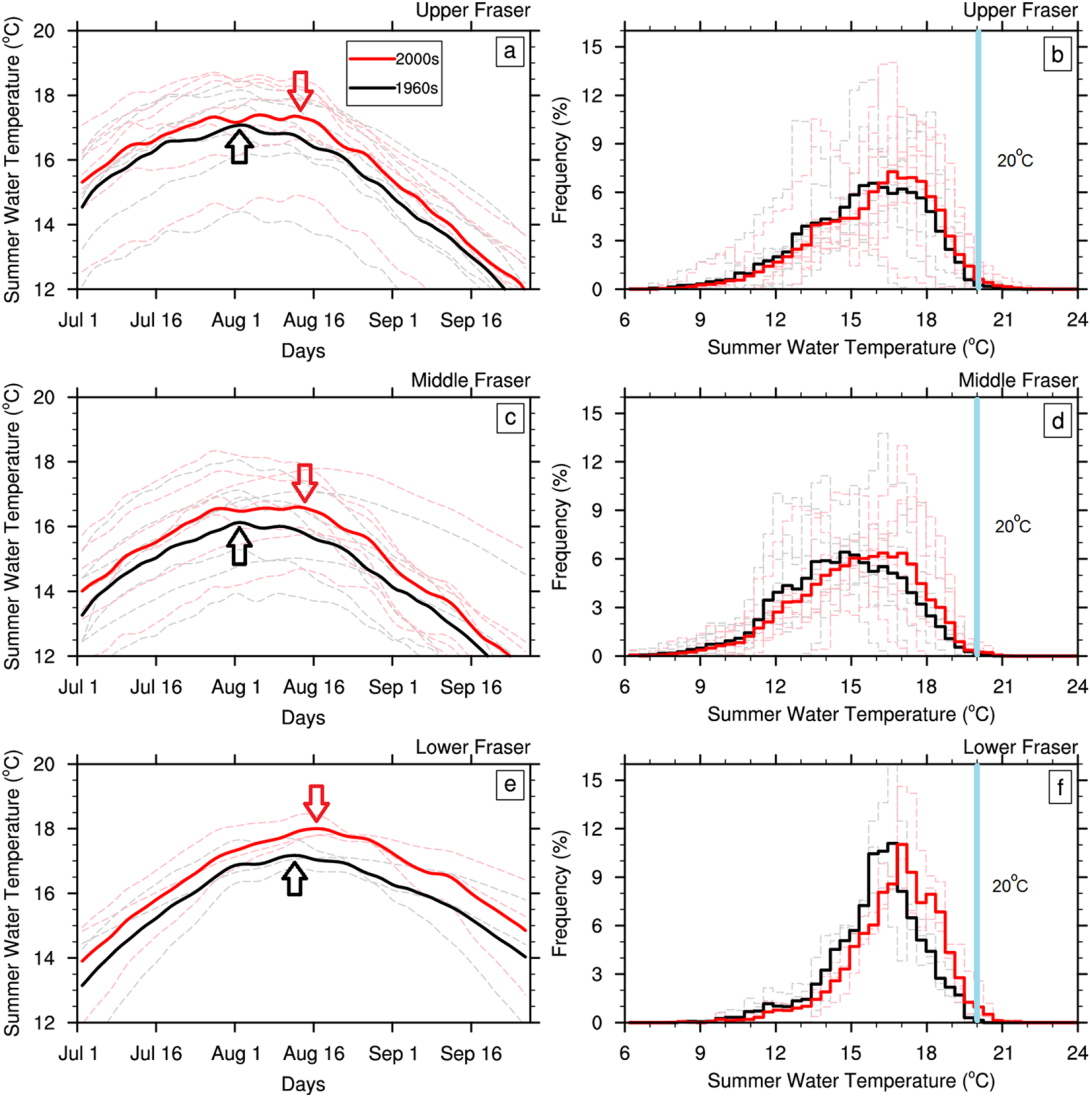
Climatology (**a,c,e**) and frequency distributions (**b,d,f**) of daily simulated summer water temperatures for the 1960s and 2000s. Climatology and distributions represent the simulated averaged response of the sites in the upper (SFJ, NFF, NCF, SGN, NVH, NIP, FSY), middle (QQL, HHY, CAC, FTC, NTR, STC, TAT) and lower (FHG, FHE, HMR) FRB, respectively. Dashed lines represent individual responses at each site. The arrows in the left column indicate days of the summer with maximum water temperature. Vertical blue lines on frequency curves represent the 20 °C critical temperature. The frequency distributions do not differ significantly at *p* < 0.05 according to a Kolmogorov‐Smirnov two‐sample test. 5-point running means smoothing was applied to all curves to facilitate comparison between time periods. See Supplementary Fig. S6 for frequency distributions at each site.

Simulated averaged responses of the summer water temperature climatology and frequency distribution in the upper, middle and lower FRB show climate change impacts on river water temperature from the 1960s to 2000s (Fig. 3). In the upper and middle FRB, the mean summer water temperatures warm by nearly 1.0 °C in the 2000s compared to the 1960s (Fig. 3a,c) with increases of ~1.5 °C in the lower FRB (Fig. 3e). In addition, the timing of the peak summer water temperature changes consistently from the 1960s to 2000s in each section of the FRB. The averaged response of water temperatures in the upper and middle FRB shows a 12-day shift in the timing of maximum summer water temperature from 2 August in the 1960s to 14 August in the 2000s, with a more modest 5-day delay in the lower FRB (from 12 August in the 1960s to 17 August in the 2000s). The timing of peak summer water temperature at individual sites closely resembles their mean response in the upper, middle and lower FRB. The temporal changes of maximum summer water temperature and corresponding day of occurrence further reveals the increasing frequency of warmer water temperature days and delay in the timing of river sites especially in the upper and middle Fraser (Supplementary Fig. S5).

The frequency distributions gradually shift toward higher summer water temperature in the recent past (i.e. 2000s) when compared to the 1960s for all three regions within the FRB with increased frequency of extreme warm events, especially those above 20 °C occurring during these times. Frequency distributions at each site show a similar pattern of a climate shift (Supplementary Fig. S6).

### Days above critical water temperatures

**Figure 4:**
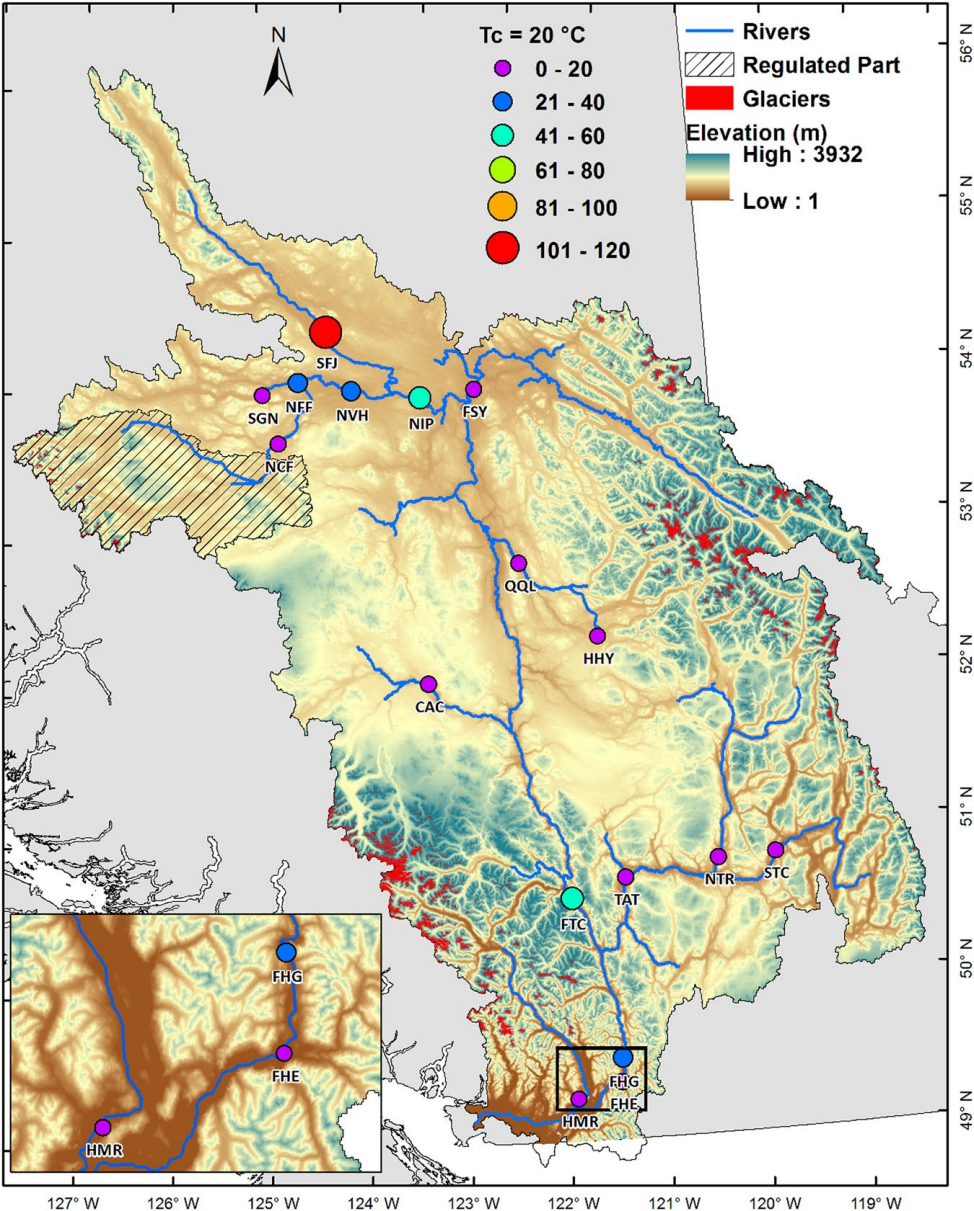
Number of days when simulated daily summer water temperatures exceeded the critical temperature of 20 °C during 1950–2015. Map generated using ESRI ArcMap 10.5.1 (http://desktop.arcgis.com/en/arcmap/).

During the 66-year study period (1950–2015), the number of days exceeding 20 °C (T_c20_) is highest across the SFJ river when compared to all other sites (Fig. 4). The changes in extreme water temperature are quite variable along the Nechako River but more modest at other FRB sites. While the water temperatures never rise beyond 18 °C (T_c18_) in the QQL and NTR rivers, most of the remaining sites show substantial increases in T_c18_ and T_c20_ occurrences. For SFJ, days exceeding T_c20_ increase markedly from 8 days in the 1960s to 71 days in the 2000s reflecting an amplifying frequency of extreme water temperatures with climate change. In contrast, at the highly regulated NVH, the largest count for days exceeding T_c18_ is in the 1960s at 100 days of which 15 days surpassed T_c20_. In the 2000s, the number of days above T_c18_ increases to 194 days with only 12 days exceeding T_c20_ (Table 2). Across the study area, day counts for all critical temperatures have increased considerably in recent years (2000s) excluding highly regulated sites (NCF and NVH) in the upper FRB and QQL and NTR in the middle FRB. The difference of T_c18_ and T_c20_ between the 2000s and the 1960s for individual months shows the maximum contribution to the total number of days above T_c20_ in summer occurs during July and August (Supplementary Table S7).

**Table 2:** Number of days when daily water temperatures exceeded critical temperatures 18 °C and 20 °C in summer.

Site	Number of days when T > T_c_ (Days)			
	18 °C		20 °C	
	1960s	2000s	1960s	2000s
SFJ	220	428	8	71
NFF	437	579	4	14
NCF	12	11	0	0
SGN	291	240	0	0
NVH	100	194	15	12
NIP	345	595	12	35
FSY	0	10	0	0
QQL	0	0	0	0
HHY	138	118	9	9
CAC	16	81	0	6
FTC	158	353	17	35
NTR	0	0	0	0
STC	263	370	0	13
TAT	98	271	0	0
FHG	223	467	0	37
FHE	128	318	0	11
HMR	121	324	0	0

See Supplementary Table S7 for individual summer months.

### Remote teleconnections

**Figure 5:**
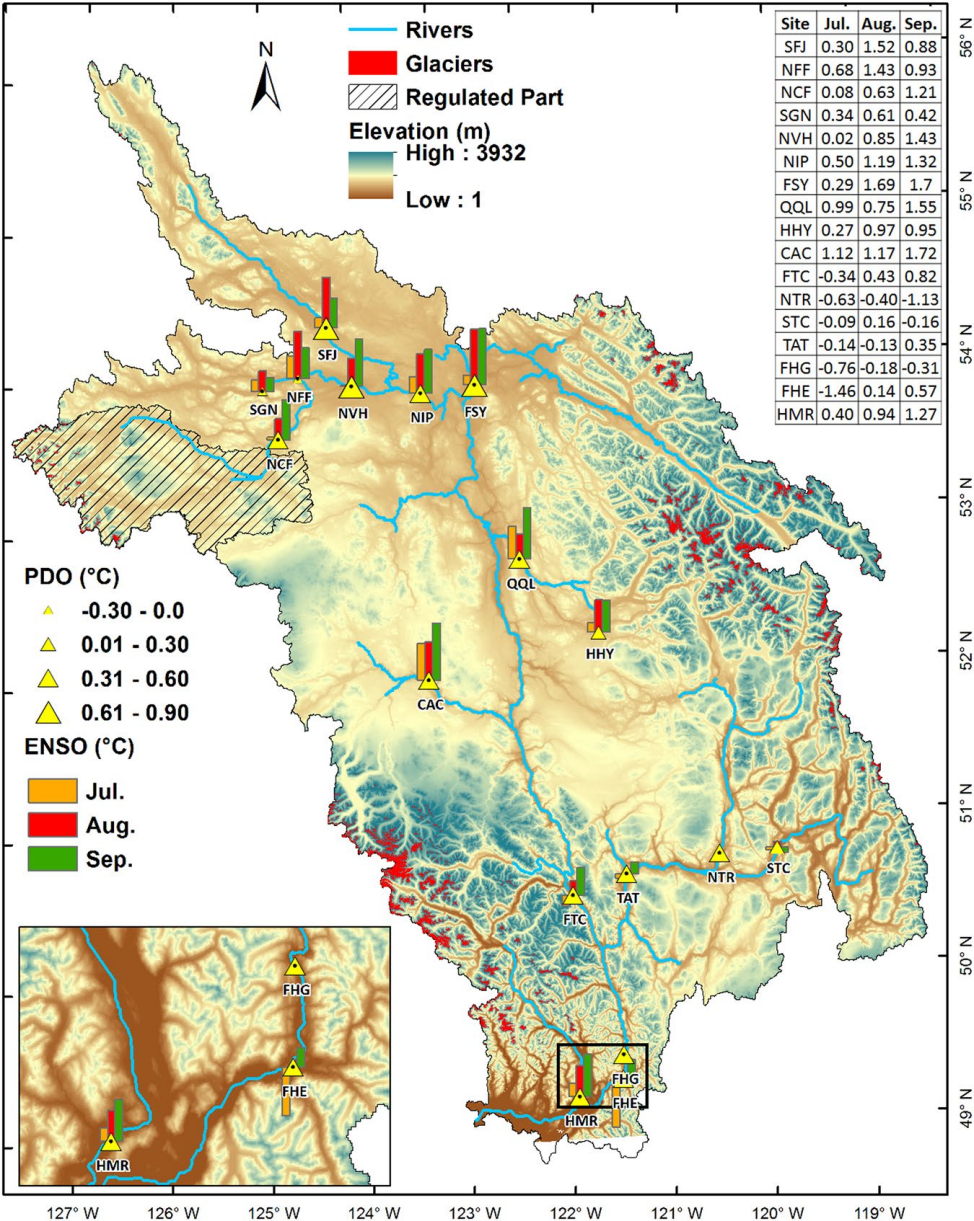
Relationships of ENSO and PDO teleconnections on simulated water temperatures. Bars show simulated water temperatures differences between the strongest 1997–98 El-Niño and 1973–74 La-Niña events. Triangles represent the magnitude of differences in summer water temperature during the positive (1977–2002) and negative (1955–1976) phases of the PDO. Triangles with black dots denote trends significant at *p* < 0.05 (computed using t-test statistics) with *p*-values provided in Supplementary Tables S8 and S9. Map generated using ESRI ArcMap 10.5.1 (http://desktop.arcgis.com/en/arcmap/).

Simulated water temperatures at all sites are associated with changes in ENSO phases (Fig. 5). Strong El-Niño and La-Niña composites show considerable variation in simulated water temperature at all sites ranging between 0.5 °C to 1.0 °C (Supplementary Table S8) with the difference between the strongest ENSO episodes (i.e. 1997–98 El-Niño minus 1973–74 La-Niña) reaching up to 2.0 °C (Fig. 5). The response to ENSO is more prominent during August coinciding with the peak up-river salmon migration period^44^. Similar to ENSO, both warm and cool PDO phases also modulate summer water temperatures. Apart from SGN and NFF, the PDO strongly affects simulated river water temperatures across all sites especially those in the upper FRB where differences between water temperatures in warm and cool PDO phases near 0.9 °C (Fig. 5, Supplementary Table S9). Overall, most of the river sites within the FRB show significant correlation between far field teleconnections and simulated river water temperatures.

### Key climatic controls of water temperatures

**Figure 6:**
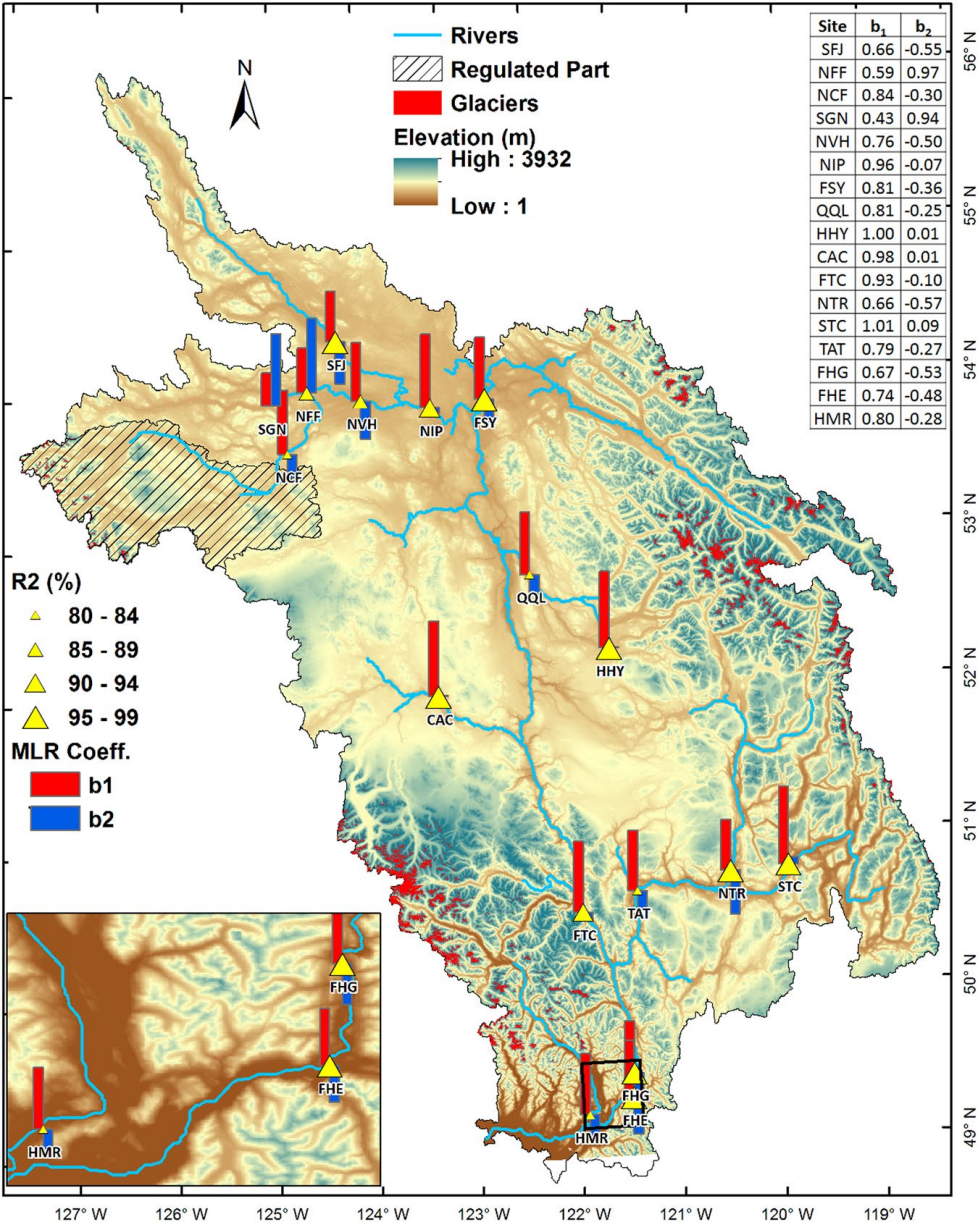
Multivariate linear regression (MLR) analysis of simulated summer water temperatures. Bars show partial regression coefficients b_1_ and b_2_ associated with air temperature and discharge, respectively. Triangles represent explained variance in %. *p*-values for coefficient b_1_ and b_2_ are provided in Supplementary Table S10. All time series are detrended prior to MLR analysis. Map generated using ESRI ArcMap 10.5.1 (http://desktop.arcgis.com/en/arcmap/).

The MLR analysis allows quantification of the contribution of mean air temperature and discharge to the simulated summer water temperature in the FRB. The variables used in the MLR analysis (Eq. 1) are, however, not completely independent of each other given the direct effect of air temperature on precipitation/snowmelt and associated discharge. Overall, the MLR performs reasonably well in estimating summer mean variability in water temperature, approaching Air2Stream simulations with explained variance (R^2^) exceeding 80% for all sites (Fig. 6). Analyses for individual summer months (July, August and September) show the MLR captures most of the explained variance in August for most sites (Supplementary Table S10).

Mean air temperature remains a key driver of changes in simulated summer water temperatures for all sites except NFF and SGN where discharge imposes a dominant control (Fig. 6). Comparison of regression coefficients b_1_ and b_2_ for individual months shows, for most sites, air temperature contributions to water temperature increase after mid-summer whereas discharge contributions decrease in late summer due to flow recession (Supplementary Fig. S7). Overall the averaged response of partial regression coefficient b_1_ during summer is 0.72, 0.88, 0.74 and 0.79 in the upper, middle, lower and whole FRB, respectively (Supplementary Table S11). Discharge, however, plays a secondary role with averaged response of the b_2_ regression coefficient equating −0.02, −0.15, −0.43 and −0.13 in the upper, middle, lower and whole FRB, respectively.

## Discussion

This study presents new insights on the evolution of daily water temperatures for 17 sites across the FRB using Air2Stream, a state-of-the-art, physically-based water temperature model. The simulations quantified changes in summer water temperature magnitudes, timing and extremes during the late 20^th^ and early 21^st^ centuries. Since the daily NSE scores ranged between 0.76–0.97 and daily RMSE and BIAS scores were reasonably low (~≤2 °C) for the calibration and validation periods, simulated water temperature diurnal and interannual variability aligned well with observations, thereby providing confidence in the Air2Stream model output. Indeed, all model performance metrics spanned the range of model reliability reported in the literature^5,21,22,27^. For example, Foreman *et al*.^21^ reported a regression model RMSE = 1.12 °C over 1953–1998 for the reconstruction of daily water temperatures at FHG. By comparison, our Air2Stream simulation RMSE = 0.98 °C at FHG thus performing equally well for the 2000–2010 calibration time period (Supplementary Table S3). Moreover, the Air2Stream model captures well associations between long-term climate oscillations such as ENSO and PDO and FRB water temperatures.

Simulated water temperatures warmed substantially across the FRB between 1950–2015 in response mainly to rising air temperatures. Robust responses of water temperatures to changes in air temperatures (e.g. at SFJ, FSY, CAC and FTC) align with the results of Isaak *et al*.^45^ for the Pacific Northwest of the United States. This effect is even amplified at SFJ owing to greater air temperature increases in northern sections of the FRB. The low gradient of elevation along the Stuart River slows the delivery of water towards the basin outlet and hence air and water temperatures remain tightly coupled. In contrast, the much steeper gradient along the Stellako and Nautley rivers decouples air and water temperatures thereby suppressing the impacts of rising air temperatures in the Air2Stream simulations at SGN and NFF. Such association of river water temperatures changes with basin characteristics is explored in many recent studies^46–49^. Overall, heightened air and river water temperature trends observed generally across parts of the Nechako watershed are well supported^50–52^ and consistent with enhanced warming effects projected for northern latitudes^53^.

Sites such as QQL, HHY, CAC, STC, TAT, FHG and FHE experience warmer conditions during summer relative to other sites (Fig. 2) and their decreasing discharge levels are likely insufficient to modulate water temperature in this season. The physical mechanism(s) however, may well be different for these sites depending on regional characteristics such as the presence of large lakes, glaciers, and gradients of elevation along waterways. For example, while discharge contributes insufficiently to simulated water temperatures at HHY, modest increases in air temperature yield lower water temperature trends relative to the neighbouring QQL site. The two branches (NTR and STC) of the Thompson River, a tributary in the southeastern section of the FRB, also exhibits dampened warming trends for river water temperature relative to other waterways, likely due to the contribution of glacier-melt water from the Monashee, Cariboo and Rocky Mountains that regulates downstream river temperatures in this system.

Consistent with Foreman *et al*.^21^, our model results reveal that ENSO and PDO strongly modulate water temperatures though atmospheric teleconnections by increasing (El-Niño and/or positive PDO phase) or decreasing (La-Niña and/or negative PDO phase) river water temperatures for most FRB sites with exception of SGN and NFF. The PDO’s relationship with SGN and NFF is most likely hindered by the presence of lakes modulating water temperatures more strongly than remote teleconnections. SGN and NFF are relatively small systems influenced by two large lakes: Francois and Fraser Lakes, respectively. SGN drains Francois Lake (254 km^2^) into Fraser Lake (55 km^2^) and NFF then drains Fraser Lake into the Nechako River. Simulation results suggest that the discharge at these two sites strongly influences water temperatures when compared to air temperature. Considering that the FRB’s river flows are strongly modulated by PDO phases^54^, it is possible that flow changes at both SGN and NFF influence the water temperatures more directly than air temperature changes weakening the relationship of PDO phases. Overall our results on ENSO/PDO relationships with simulated water temperatures provide guidance on measures of predictability for seasonal anomalies of river water temperatures in the FRB. Improving understanding of these far-field relationships with changes in water temperature is crucial for the future conservation of different aquatic species, especially salmon populations.

Changing aquatic conditions have implications for migrating salmon in the FRB as the mortality increases markedly when water temperatures surpass critical temperatures (e.g. 18 °C)^28^. The frequency of critical temperatures has amplified appreciably in recent decades (e.g. 2000s), suggesting potential increasing stress on migratory salmon^55^. Perhaps surprisingly, the northernmost river system (SFJ) experiences the highest frequency of critical water temperatures due to amplified atmospheric warming in the northern FRB. Further, with a nearly 1.0 °C rise in mean summer water temperature during 1950–2015, days exceeding critical temperatures at many sites within the FRB have more than doubled particularly for days surpassing T_c20_ with a few exceptions across the FRB. Our simulated increases in mean summer water and critical temperatures are consistent with past studies focusing on Pacific salmon populations in the Columbia and Fraser river systems^56–58^. Studies^56,59^ have indeed reported significant increases in summer freshwater temperatures since the 1950s during spawning migrations in the Columbia and Fraser rivers, thus posing a major threat to the future sustainability of salmon populations in these systems^40,60^.

An exception to increasing days exceeding critical temperatures occurs in the Nechako River in the northwest portion of the FRB. The Nechako River has been regulated by Alcan (now owned by Rio Tinto) since the construction of the Kenney Dam, the Skins Lake Spillway, and the Nechako Reservoir in the 1950s^61,62^. Summer flows in the Nechako River are further regulated in response to forecasted air temperatures owing to a 1980 court injunction^42,50,63^. Following the court ruling, Rio Tinto implemented the STMP in 1981 to suppress river water temperatures to <20 °C for the Nechako River at Finmoore (~40 km downstream from NVH) from 20 July to 20 August through controlled releases from Skins Lake Spillway for the benefit of migrating salmon. The Air2Stream model, forced with regulated discharge, simulates reasonably well the impact of the STMP on Nechako River water temperatures, including the NVH site. Model results suggest that the number of days when simulated water temperatures rise above T_c20_ has not changed during the pre- and post-STMP time periods despite rising air temperatures, thereby revealing the effectiveness of the STMP in meeting temperature requirements near NVH. The warming trends in the Nechako River are likely not related to the impact of regulation, given that the varying summer water temperature response to climate change in its unregulated tributaries, i.e. strong warming summer trend at SFJ and insignificant summer trends at NFF and SGN. We tested the impact of regulation on water temperatures using Air2Stream sensitivity runs for NVH. Regulation decreases water temperatures especially during the STMP time window of 20 July to 20 August whereas the long-term trend abates due to regulation. This implies that overall water temperature increases in the Nechako River are mainly due to climate change rather than regulation.

This study’s results rely on Air2Stream simulations subjected to calibration of model parameters. Available water temperature records remain limited for many FRB river sites posing challenges in calibrating and validating the Air2Stream model. The available water temperature records also have data gaps and random, spurious data entries which, to some extent, are rectified by quality control and analysis. Another limitation of the Air2Stream model application is its assumption of a stationary response between air temperature, daily discharge and simulated water temperature. Furthermore, the model cannot adequately account for all the complexities of river thermal properties, especially the effects of different surface radiative and energy fluxes. Other processes affecting river water temperatures including groundwater exfiltration, lake and river ice melting, and cold water releases from the hypolimnion of upstream lakes are also perhaps not well captured by Air2Stream. Finally, the ability of the Air2Stream model to account for the impacts of land use changes directly into its simulation remains limited, although variations in river runoff due to land cover changes (e.g. forest harvesting leading to less vegetative water demands and hence higher runoff) are indirectly included in model simulations through discharge forcings. Nevertheless, the study provides valuable insights into the historical thermal state of 17 river sites across the FRB, which will prove useful for developing future water management strategies that may affect aquatic resources such as salmon.

## Conclusion

This study formulates an important baseline for the historical changes in the simulated water temperatures across the FRB using the Air2Stream model forced with observational datasets. A key finding arising from this work is the model’s ability to reproduce observed interannual variability in summer water temperature for 17 rivers sites across the FRB. Whereas previous studies typically have implemented and evaluated statistical models at a limited number of river sites, this effort uses the physically-based Air2Stream model to simulate consistent and reliable estimates of daily water temperatures across the entire FRB. Our model results provide useful guidance for fisheries management for future governance and decision-making in the FRB. Climate change may continue to impose effects on salmon via increases in instream mortality and/or challenges to migration due to alterations in discharge. The ongoing warming of river thermal conditions in the FRB indicate that further efforts are needed to investigate projected changes in water temperatures under future climate change. Our future efforts will look at potential future water temperatures across the FRB using a combination of the Air2Stream model, future climate projections and projected discharge outputs available through a hydrological model used in our previous efforts^43,64^.

## Supplementary information

Supplementary Information
