# Supplementary material for: Modelling the impacts of climate change on riverine thermal regimes in western Canada’s largest Pacific watershed
Source: Sci Rep. 2019 Aug 6;9:11398. doi: 10.1038/s41598-019-47804-2 (PMC6684650; doi:10.1038/s41598-019-47804-2)
Supplement: Supplementary file 1 — Supplementary Information [file 41598_2019_47804_MOESM1_ESM.docx]

**Modelling the impacts of climate change on riverine thermal regimes in western Canada’s largest Pacific watershed**

Siraj Ul Islam*, Rachel W. Hay, Stephen J. Déry and Barry P. Booth

Environmental Science and Engineering Program, University of Northern British Columbia, Prince George, British Columbia, Canada

Manuscript SREP-19-10001

Submitted in revised form to *Scientific Reports*

July 15^th^, 2019

**Supplementary Information**

Corresponding Author: Siraj Ul Islam

Environmental Science and Engineering Program

University of Northern British Columbia

3333 University Way

Prince George, BC, Canada, V2N 4Z9

E-mail: sirajul.islam@unbc.ca

**Introduction**

This file contains additional explanatory information, supplementary figures and supplementary tables as referenced in the main paper. Supplementary Tables S1 and S2 provide detailed information on observed data collection, data availability, locations and sources for the water temperature and river discharge variables used in Air2Stream simulations.

**Construction of forcing dataset for the Air2Stream model:**

Observed water temperature data for 17 sites were obtained from different sources that include the Water Survey of Canada (WSC), Triton Environmental Consultants, Rio Tinto and the Department of Fisheries and Oceans (DFO) Canada. For several river sites, daily water temperature time series from multiple sources were merged to fill temporal gaps (Supplementary Table S1).

Observed daily discharge data were acquired from Water Survey of Canada measurement locations (Supplementary Table S2) publicly available at <https://wateroffice.ec.gc.ca/search/historical_e.html>. For some sites, discharge time series from different sources were combined to fill extended temporal gaps or, if data were unavailable, data from the nearest site were used. For NCF, the discharge data were merged from the Nechako River below Cheslatta Falls (08JA017) and Skins Lake Spillway at Nechako Reservoir (08JA013) gauging stations. The 1955-1979 daily discharge data for the Skins Lake Spillway at Nechako Reservoir were combined with Nechako River below Cheslatta Falls to cover the data gap prior to 1980. To fill in the 1950-1954 data gap, daily values from the 1955-1960 climatology were used. For the Horsefly River above McKinley Creek river site, data were summed for the Horsefly River above McKinley Creek (08KH010), McKinley Creek below outlet of McKinley Lake (08KH020), and Moffat Creek near Horsefly (08KH019) for the overlapping period of 1964-2015. The 1950-1958 time period was extracted from the Horsefly River at Horsefly (08KH007) and the 1959-1963 gap was filled with the averaged climatology data (1950-1958) from Horsefly River at Horsefly (08KH007). Discharge data for the Fraser River at Hells Gate (FHG) were not available and therefore discharge data from the Fraser River at Hope were used to drive the Air2Stream model for this site. These are two nearby locations approximately 20 km apart, with no major tributaries joining the Fraser River between the two sites (Fig. 1 in the main text).

**Supplementary Table S1.** List of observed water temperature databases, by data provider and location. Data sources include the Water Survey of Canada (WSC), Triton Environmental Consultants, Rio Tinto and the Department of Fisheries and Oceans (DFO) Canada. For the sites with an asterisk, data were merged from WSC and DFO except NFF where data were merged from WSC and Triton/Rio Tinto.

| **Site** | **Data Provider** | **Latitude (**°N) | **Longitude** (°W) | **Seasonal/**  **Annual** | **Data Record** |
| --- | --- | --- | --- | --- | --- |
| SFJ | DFO | 54.41 | 124.27 | Seasonal | 1981-2009 |
| NFF* | DFO/Triton/Rio Tinto | 54.08 | 124.60 | Seasonal/Annual | 1981-2015 |
| NCF | Triton/Rio Tinto | 53.68 | 124.83 | Seasonal/Annual | 2001-2015 |
| SGN | WSC (station 08JB002) | 54.00 | 125.00 | Annual | 2011-2015 |
| NVH* | DFO/WSC (station 08JC001) | 54.02 | 124.00 | Seasonal/Annual | 1980-2015 |
| NIP* | DFO/WSC (station 08JC002) | 53.96 | 123.23 | Seasonal/Annual | 1981-2015 |
| FSY* | DFO/WSC (Station 08KB001) | 54.00 | 122.62 | Seasonal (to 2005), Annual afterward | 1995-2015 |
| QQL* | DFO/WSC (Station 08KH006) | 52.84 | 122.22 | Seasonal (to 2005), Annual afterward | 1995-2015 |
| HHY | DFO | 52.33 | 121.41 | Seasonal | 1995-2005 |
| CAC* | DFO/WSC (Station 08MB005) | 52.07 | 123.26 | Seasonal (to 2005), Annual afterward | 1995-2015 |
| FTC | WSC (Station 08MF040) | 50.61 | 121.85 | Annual | 2006-2015 |
| NTR | DFO | 50.82 | 120.30 | Seasonal (to 2005), Annual afterward | 1995-2015 |
| STC* | DFO/WSC (Station 08LE031) | 50.83 | 119.70 | Seasonal | 1995-2015 |
| TAT* | DFO/WSC (Station 08LF051) | 50.73 | 121.28 | Seasonal (to 2005), Annual afterward | 1995-2005;  2006-2015 |
| FHG | DFO | 49.54 | 121.43 | Annual | 1950-2015 |
| FHE | WSC (Station 08MF005) | 49.38 | 121.45 | Annual | 2008-2015 |
| HMR | WSC (Station  08MG022) | 49.28 | 121.90 | Annual | 2013-2015 |

**Supplementary Table S2.** List of Water Survey of Canada (WSC) hydrometric gauges for which daily discharge data were used in the water temperature simulations. Data availability is only shown for the time period of this study, i.e. 1950-2015. For the sites with an asterisk, data were merged from multiple gauges.

| **Site** | **Gauges ID** | **Hydrometric Gauge Name** | **Latitude**  **(**°N) | **Longitude**  (°W) | **Data Availability** |
| --- | --- | --- | --- | --- | --- |
| SFJ | 08JE001 | Stuart River near Fort St. James | 54.42 | 124.27 | 1950-2015 |
| NFF | 08JB003 | Nautley River near Fort Fraser | 54.08 | 124.59 | 1950-2015 |
| NCF* | 08JA017 | Skins Lake Spillway at Nechako Reservoir | 53.77 | 125.97 | 1956-1979 |
|  | 08JA013 | Nechako River below Cheslatta Falls | 53.68 | 124.83 | 1980-2015 |
| SGN | 08JB002 | Stellako River at Glenannan | 54.01 | 125.00 | 1950-2015 |
| NVH | 08JC001 | Nechako River at Vanderhoof | 54.02 | 124.00 | 1950-2015 |
| NIP | 08JC002 | Nechako River at Isle Pierre | 53.96 | 123.23 | 1950-2015 |
| FSY | 08KB001 | Fraser River at Shelley | 54.01 | 122.62 | 1950-2015 |
| QQL | 08KH006 | Quesnel River near Quesnel | 52.84 | 122.22 | 1950-2015 |
| HHY* | 08KH010 | Horsefly River above McKinley Creek | 52.29 | 121.06 | 1964-2015 |
|  | 08KH020 | McKinley Creek below outlet of McKinley Lake | 52.28 | 121.00 | 1964-2015 |
|  | 08KH019 | Moffat Creek near Horsefly | 52.31 | 121.40 | 1964-2015 |
|  | 08KH007 | Horsefly River at Horsefly | 52.37 | 121.31 | 1950-1958 |
| CAC | 08MB005 | Chilcotin River below Big Creek | 51.84 | 122.65 | 1970-2015 |
| FTC | 08MF040 | Fraser River above Texas Creek | 50.61 | 121.85 | 1951-2015 |
| NTR | 08LB064 | North Thompson River at McLure | 51.04 | 120.24 | 1958-2015 |
| STC | 08LE031 | South Thompson River at Chase | 50.76 | 119.74 | 1950-2015 |
| TAT | 08LF051 | Thompson River near Spences Bridge | 50.35 | 121.39 | 1950-2015 |
| FHG | 08MF005 | Fraser River at Hope | 49.38 | 121.45 | 1950-2015 |
| FHE | 08MF005 | Fraser River at Hope | 49.38 | 121.45 | 1950-2015 |
| HMR | 08MG022 | Harrison River below Morris Creek | 49.28 | 121.90 | 1951-2015 |

**Supplementary Table S3.** Air2Stream model calibration performance metrics. The daily scores were computed as per availability of daily data (summer only or whole year) used for model calibration (see Supplementary Table S1 for details). Summer mean scores were computed using the annual water temperatures averaged over summer seasons.

| **Site** | **Time Period** | **Daily** | | | **Interannual**  **Summer Mean** | |
| --- | --- | --- | --- | --- | --- | --- |
|  |  | **NSE** | **RMSE (°C)** | **BIAS (°C)** | **RMSE (°C)** | **BIAS (°C)** |
| SFJ | 1995-2004 | 0.89 | 0.85 | -0.01 | 0.32 | 0.02 |
| NFF | 2009-2015 | 0.96 | 1.27 | 0.04 | 1.04 | -0.09 |
| NCF | 2002-2011 | 0.96 | 1.18 | 0.23 | 0.59 | 0.09 |
| SGN | 2009-2015 | 0.94 | 1.43 | 0.04 | 1.14 | 0.60 |
| NVH | 2002-2011 | 0.97 | 1.07 | 0.28 | 0.48 | -0.12 |
| NIP | 2006-2015 | 0.97 | 1.19 | 0.10 | 0.59 | 0.12 |
| FSY | 2006-2015 | 0.96 | 1.01 | 0.02 | 0.33 | -0.16 |
| QQL | 2006-2015 | 0.94 | 1.42 | 0.10 | 0.48 | 0.27 |
| HHY | 1995-2004 | 0.81 | 1.30 | -0.01 | 0.35 | 0.05 |
| CAC | 2006-2015 | 0.95 | 1.35 | -0.02 | 0.39 | -0.21 |
| FTC | 2006-2015 | 0.96 | 0.98 | 0.11 | 0.52 | 0.09 |
| NTR | 2006-2015 | 0.96 | 1.10 | 0.07 | 1.11 | 0.14 |
| STC | 1995-2004 | 0.76 | 1.21 | 0.00 | 0.64 | 0.03 |
| TAT | 2006-2015 | 0.88 | 2.04 | 0.07 | 1.07 | 0.85 |
| FHG | 2000-2010 | 0.86 | 0.98 | 0.09 | 0.68 | 0.20 |
| FHE | 2009-2015 | 0.90 | 2.01 | -0.06 | 0.73 | 0.15 |
| HMR | 2009-2015 | 0.93 | 1.00 | 0.13 | 0.77 | 0.65 |
| **Mean** | | 0.92 | 1.26 | 0.07 | 0.66 | 0.13 |
| **Min.** | | 0.76 | 0.85 | -0.06 | 0.32 | -0.21 |
| **Max.** | | 0.97 | 2.04 | 0.28 | 1.14 | 0.85 |

**Supplementary Table S4.** Same as Supplementary Table S3 but for Air2Stream model validation. Model performance metrics were only computed for sites having at least five years of observed data for validation.

| **Site** | **Time Period** | **Daily** | | | **Interannual**  **Summer Mean** | |
| --- | --- | --- | --- | --- | --- | --- |
|  |  | **NSE** | **RMSE (°C)** | **BIAS (°C)** | **RMSE (°C)** | **BIAS (°C)** |
| SFJ | 1985-1994 | 0.80 | 1.09 | 0.28 | 1.65 | 1.22 |
| NFF | 2000-2008 | 0.96 | 1.41 | -0.22 | 0.65 | -0.04 |
| NCF | 2012-2015 | 0.93 | 1.59 | 1.05 | 0.75 | 0.67 |
| SGN | - | - | - | - | - | - |
| NVH | 2012-2015 | 0.97 | 1.11 | 0.05 | 0.26 | -0.11 |
| NIP | - | - | - | - | - | - |
| FSY | 2000-2005 | 0.96 | 1.16 | -0.12 | 0.30 | -0.15 |
| QQL | 2000-2005 | 0.95 | 1.32 | -0.16 | 0.97 | 0.50 |
| HHY | - | - | - | - | - | - |
| CAC | 2000-2005 | 0.92 | 1.60 | -0.36 | 0.70 | -0.58 |
| FTC | - | - | - | - | - | - |
| NTR | 2000-2005 | 0.94 | 1.34 | 0.79 | 1.53 | 1.52 |
| STC | - | - | - | - | - | - |
| TAT | - | - | - | - | - | - |
| FHG | 2011-2015 | 0.88 | 2.16 | 1.65 | 0.54 | 0.22 |
| FHE | - | - | - | - | - | - |
| HMR | - | - | - | - | - | - |
| **Mean** | | 0.92 | 1.42 | 0.33 | 0.82 | 0.36 |
| **Min.** | | 0.80 | 1.09 | -0.36 | 0.26 | -0.58 |
| **Max.** | | 0.97 | 2.16 | 1.65 | 1.65 | 1.52 |

**Supplementary Table S5.** Simulated water temperature trends for summer months and summer means. Bold values denote trends significant at *p* < 0.05 computed using t-test statistics. The last two rows provide the mean and standard deviation (SD) of trends for all sites. Grey shading denotes regulated sites.

| **Site** | **Water Temperature Trend (°C (66 yr)^-1^)** | | | | | | | |
| --- | --- | --- | --- | --- | --- | --- | --- | --- |
|  | **Jul.** | ***p*-value** | **Aug.** | ***p*-value** | **Sep.** | ***p*-value** | **Summer** | ***p*-value** |
| SFJ | **1.21** | < 0.001 | **1.68** | < 0.001 | **1.57** | < 0.001 | **1.40** | < 0.001 |
| NFF | 0.27 | 0.12 | 0.43 | 0.12 | 0.22 | 0.55 | 0.27 | 0.18 |
| NCF | **1.29** | < 0.001 | **0.68** | < 0.001 | 0.24 | 0.41 | **0.71** | < 0.001 |
| SGN | -0.05 | 0.69 | -0.07 | 0.78 | -0.50 | 0.13 | -0.19 | 0.39 |
| NVH | **1.12** | < 0.001 | **1.10** | < 0.001 | **1.33** | < 0.001 | **1.17** | < 0.001 |
| NIP | **0.79** | < 0.001 | **1.03** | < 0.001 | 0.75 | 0.05 | **0.84** | < 0.001 |
| FSY | **1.09** | 0.01 | **1.69** | < 0.001 | 0.98 | 0.05 | **1.21** | < 0.001 |
| QQL | **1.24** | < 0.001 | **1.37** | < 0.001 | **0.87** | 0.01 | **1.17** | < 0.001 |
| HHY | 0.45 | 0.21 | 0.52 | 0.21 | 0.01 | 0.99 | 0.31 | 0.21 |
| CAC | **1.75** | < 0.001 | **1.80** | < 0.001 | 0.87 | 0.12 | **1.45** | < 0.001 |
| FTC | **1.56** | < 0.001 | **1.93** | < 0.001 | **1.23** | 0.02 | **1.56** | < 0.001 |
| NTR | **0.81** | 0.02 | **1.24** | < 0.001 | **1.18** | < 0.001 | **1.06** | < 0.001 |
| STC | **0.42** | 0.01 | **0.70** | 0.01 | 0.35 | 0.27 | **0.45** | < 0.001 |
| TAT | **1.01** | < 0.001 | **1.31** | < 0.001 | **1.14** | < 0.001 | **1.12** | < 0.001 |
| FHG | **0.94** | 0.01 | **1.37** | < 0.001 | **1.29** | 0.01 | **1.13** | < 0.001 |
| FHE | **1.43** | 0.02 | **1.50** | 0.01 | **1.27** | < 0.001 | **1.35** | < 0.001 |
| HMR | **0.94** | 0.01 | **1.21** | < 0.001 | **1.11** | < 0.001 | **1.00** | < 0.001 |
| **Mean** | 0.96 | - | 1.15 | - | 0.82 | - | 0.94 | - |
| **SD** | 0.46 | - | 0.53 | - | 0.55 | - | 0.47 | - |

**Supplementary Table S6.** Simulated summer water temperature interannual variability (standard deviation, SD) and corresponding signal to noise ratio (SNR). Bold values denote SNR significant at *p* < 0.05 computed using t-test statistics. Grey shading denotes regulated sites. Mann-Kendall Test (MKT) represents monotonic trends.

| **Site** | **SD (°C)** | **SNR (MKT/SD)** |
| --- | --- | --- |
| SFJ | 0.84 | **1.67** |
| NFF | 0.49 | 0.55 |
| NCF | 0.45 | **1.57** |
| SGN | 0.45 | -0.42 |
| NVH | 0.66 | **1.77** |
| NIP | 0.50 | **1.68** |
| FSY | 0.78 | **1.55** |
| QQL | 0.70 | **1.67** |
| HHY | 0.57 | 0.54 |
| CAC | 0.81 | **1.79** |
| FTC | 0.88 | **1.77** |
| NTR | 0.65 | **1.63** |
| STC | 0.37 | **1.22** |
| TAT | 0.66 | **1.70** |
| FHG | 0.70 | **1.61** |
| FHE | 0.87 | **1.55** |
| HMR | 0.66 | **1.52** |

**Supplementary Table S7.** Difference in the number of days in summer months when daily water temperatures exceeded critical temperatures (T_c_) during the 2000s compared to the 1960s. The counts for 18**°**C are not independent of those for 20**°**C.

| **Site** | **Number of days when**  **T > T**_c_  **2000s - 1960s (Days)** | | | | | | | |
| --- | --- | --- | --- | --- | --- | --- | --- | --- |
|  | **T_c_ = 18°C** | | | | **T_c_ = 20°C** | | | |
|  | **Jul.** | **Aug.** | **Sep.** | **Summer** | **Jul.** | **Aug.** | **Sep.** | **Summer** |
| **SFJ** | 102 | 102 | 4 | 208 | 17 | 46 | 0 | 63 |
| **NFF** | 69 | 73 | 0 | 142 | 3 | 7 | 0 | 10 |
| **NCF** | 3 | -8 | 4 | -1 | 0 | 0 | 0 | 0 |
| **SGN** | -24 | -22 | -5 | -51 | 0 | 0 | 0 | 0 |
| **NVH** | 49 | 25 | 20 | 94 | 1 | -4 | 0 | -3 |
| **NIP** | 151 | 92 | 7 | 250 | 11 | 12 | 0 | 23 |
| **FSY** | 2 | 8 | 0 | 10 | 0 | 0 | 0 | 0 |
| **QQL** | 0 | 0 | 0 | 0 | 0 | 0 | 0 | 0 |
| **HHY** | 0 | -18 | -2 | -20 | 5 | -5 | 0 | 0 |
| **CAC** | 27 | 38 | 0 | 65 | 3 | 3 | 0 | 6 |
| **FTC** | 66 | 115 | 14 | 195 | 8 | 10 | 0 | 18 |
| **NTR** | 0 | 0 | 0 | 0 | 0 | 0 | 0 | 0 |
| **STC** | 37 | 69 | 1 | 107 | 6 | 7 | 0 | 13 |
| **TAT** | 21 | 130 | 22 | 173 | 0 | 0 | 0 | 0 |
| **FHG** | 54 | 147 | 43 | 244 | 7 | 24 | 6 | 37 |
| **FHE** | 11 | 122 | 57 | 190 | 1 | 10 | 0 | 11 |
| **HMR** | 17 | 125 | 61 | 203 | 0 | 0 | 0 | 0 |

**Supplementary Table S8.** Simulated water temperature differences (Diff.) between composites of strong El-Niño (1957-58, 1965-66, 1972-73, 1982-83, 1987-88 1991-92, 1997-98, 2015-16) and strong La-Niña (1973-74, 1975-76, 1988-89, 1998-99, 1999-2000, 2007-08, 2010-11, 2011-12) years. Strong El-Niño and La-Niña events were selected based on the 3-month running mean sea surface temperature (SST) anomaly for the Niño 3.4 region (5^o^N-5^o^S, 170^o^W-120^o^W) in the tropical Pacific Ocean (NOAA, Climate prediction center, https://origin.cpc.ncep.noaa.gov/). Bold values denote differences significant at *p* < 0.05 computed using t-test statistics.

| **Site** | **Jul.** | | **Aug.** | | **Sep.** | |
| --- | --- | --- | --- | --- | --- | --- |
|  | **Diff. (°C)** | ***p*-value** | **Diff. (°C)** | ***p*-value** | **Diff. (°C)** | ***p*-value** |
| SFJ | 0.04 | 0.76 | **0.41** | < 0.001 | 0.02 | 0.93 |
| NFF | **-0.17** | 0.04 | -0.03 | 0.72 | -0.26 | 0.07 |
| NCF | 0.05 | 0.64 | **0.14** | 0.02 | 0.00 | 0.97 |
| SGN | 0.00 | 0.98 | 0.04 | 0.63 | -0.14 | 0.22 |
| NVH | 0.04 | 0.70 | **0.26** | 0.01 | 0.00 | 1.00 |
| NIP | -0.03 | 0.72 | **0.26** | 0.01 | 0.01 | 0.94 |
| FSY | -0.11 | 0.40 | 0.16 | 0.21 | -0.19 | 0.25 |
| QQL | 0.16 | 0.13 | -0.08 | 0.35 | -0.13 | 0.17 |
| HHY | -0.26 | 0.10 | 0.14 | 0.36 | -0.19 | 0.37 |
| CAC | -0.14 | 0.36 | -0.23 | 0.12 | **-0.43** | 0.01 |
| FTC | -0.10 | 0.53 | **-0.44** | < 0.001 | **-0.82** | < 0.001 |
| NTR | 0.06 | 0.58 | -0.19 | 0.05 | **-0.91** | < 0.001 |
| STC | -0.19 | 0.05 | -0.14 | 0.16 | **-0.46** | < 0.001 |
| TAT | **0.24** | 0.01 | **-0.28** | < 0.001 | **-0.41** | < 0.001 |
| FHG | 0.07 | 0.53 | -0.06 | 0.53 | **-0.86** | < 0.001 |
| FHE | 0.11 | 0.56 | -0.05 | 0.62 | **-0.68** | < 0.001 |
| HMR | **0.58** | < 0.001 | **0.23** | < 0.001 | -0.05 | 0.44 |

**Supplementary Table S9.** Simulated water temperature differences between the positive (1977-2002) and negative (1955-1976) phases of the Pacific Decadal Oscillation (PDO). Bold values denote differences significant at *p* < 0.05 computed using t-test statistics.

| **Site** | **+PDO**  **(°C)** | **-PDO**  **(°C)** | **Difference**  **(°C)** | ***p*-value** |
| --- | --- | --- | --- | --- |
| **SFJ** | 16.06 | 15.21 | **0.85** | < 0.001 |
| **NFF** | 16.42 | 16.56 | **-0.14** | 0.02 |
| **NCF** | 15.34 | 14.99 | **0.36** | < 0.001 |
| **SGN** | 16.21 | 16.53 | **-0.32** | < 0.001 |
| **NVH** | 15.66 | 14.98 | **0.69** | < 0.001 |
| **NIP** | 16.42 | 15.95 | **0.47** | < 0.001 |
| **FSY** | 12.62 | 11.93 | **0.69** | < 0.001 |
| **QQL** | 14.21 | 13.78 | **0.42** | < 0.001 |
| **HHY** | 14.08 | 14.00 | 0.08 | 0.36 |
| **CAC** | 13.80 | 13.21 | **0.59** | < 0.001 |
| **FTC** | 15.44 | 14.94 | **0.50** | < 0.001 |
| **NTR** | 12.74 | 12.42 | **0.32** | < 0.001 |
| **STC** | 15.85 | 15.77 | 0.08 | 0.20 |
| **TAT** | 16.16 | 15.85 | **0.32** | < 0.001 |
| **FHG** | 16.20 | 15.72 | **0.48** | < 0.001 |
| **FHE** | 15.43 | 15.02 | **0.41** | < 0.001 |
| **HMR** | 16.52 | 16.16 | **0.36** | < 0.001 |

**Supplementary Table S10.** Explained variance (R^2^) of the multivariate linear regression (MLR) analysis (see eq. 1 in the main text) for summer months. The *p*-values, computed using t-test statistics, for regression coefficient b_2_ (discharge) are provided for summer means and individual months. *p-*values for b_1_ (mean temperature) are all significant and < 0.001.

| **Site** | **R^2^ (%)** | | | ***p*-value for regression coefficient b_2_** | | | |
| --- | --- | --- | --- | --- | --- | --- | --- |
|  | **Jul.** | **Aug.** | **Sep.** | **Summer** | **Jul.** | **Aug.** | **Sep.** |
| SFJ | 95.43 | 95.87 | 97.33 | < 0.001 | < 0.001 | < 0.001 | < 0.001 |
| NFF | 89.17 | 90.46 | 92.35 | < 0.001 | < 0.001 | < 0.001 | < 0.001 |
| NCF | 81.40 | 85.64 | 84.51 | < 0.001 | < 0.001 | < 0.001 | < 0.001 |
| SGN | 30.91 | 94.36 | 91.52 | < 0.001 | < 0.001 | < 0.001 | < 0.001 |
| NVH | 85.31 | 86.63 | 88.51 | < 0.001 | < 0.001 | < 0.001 | 0.20 |
| NIP | 88.77 | 93.26 | 91.02 | 0.03 | < 0.001 | 0.01 | 0.47 |
| FSY | 95.51 | 93.60 | 91.59 | < 0.001 | < 0.001 | < 0.001 | < 0.001 |
| QQL | 62.82 | 71.99 | 62.29 | < 0.001 | < 0.001 | < 0.001 | < 0.001 |
| HHY | 96.02 | 96.99 | 97.60 | 0.37 | 0.22 | 0.87 | 1.00 |
| CAC | 88.08 | 88.44 | 89.16 | 0.81 | 0.66 | 0.93 | 0.88 |
| FTC | 81.73 | 80.61 | 80.34 | 0.01 | 0.03 | 0.03 | 0.02 |
| NTR | 96.51 | 94.33 | 95.44 | < 0.001 | < 0.001 | < 0.001 | < 0.001 |
| STC | 95.64 | 97.12 | 98.04 | < 0.001 | 0.77 | 0.31 | 0.06 |
| TAT | 65.35 | 72.50 | 55.59 | < 0.001 | < 0.001 | < 0.001 | < 0.001 |
| FHG | 91.95 | 90.88 | 92.54 | < 0.001 | < 0.001 | < 0.001 | < 0.001 |
| FHE | 92.19 | 83.43 | 74.02 | < 0.001 | < 0.001 | < 0.001 | < 0.001 |
| HMR | 63.60 | 63.11 | 31.70 | < 0.001 | < 0.001 | < 0.001 | 0.45 |

**Supplementary Table S11.** Averaged response of MLR partial regression coefficients (see eq. 1 in the main text) over all 17 sites (referred to as the whole FRB). These 17 sites were further averaged into three regions: upper (SFJ, NFF, NCF, SGN, NVH, NIP, FSY), middle (QQL, HHY, CAC, FTC, NTR, STC, TAT) and lower (FHG, FHE, HMR) FRB.

| **Region** | **b_1_** | | | | **b_2_** | | | |
| --- | --- | --- | --- | --- | --- | --- | --- | --- |
|  | **Summer** | **Jul.** | **Aug.** | **Sep.** | **Summer** | **Jul.** | **Aug.** | **Sep.** |
| Upper FRB | 0.72 | 0.69 | 0.80 | 0.81 | 0.02 | -0.18 | 0.02 | 0.15 |
| Middle FRB | 0.88 | 0.80 | 0.84 | 0.86 | -0.15 | -0.20 | -0.19 | -0.18 |
| Lower FRB | 0.74 | 0.66 | 0.70 | 0.70 | -0.43 | -0.47 | -0.43 | -0.28 |
| Whole FRB | 0.79 | 0.73 | 0.80 | 0.81 | -0.13 | -0.24 | -0.14 | -0.06 |

| **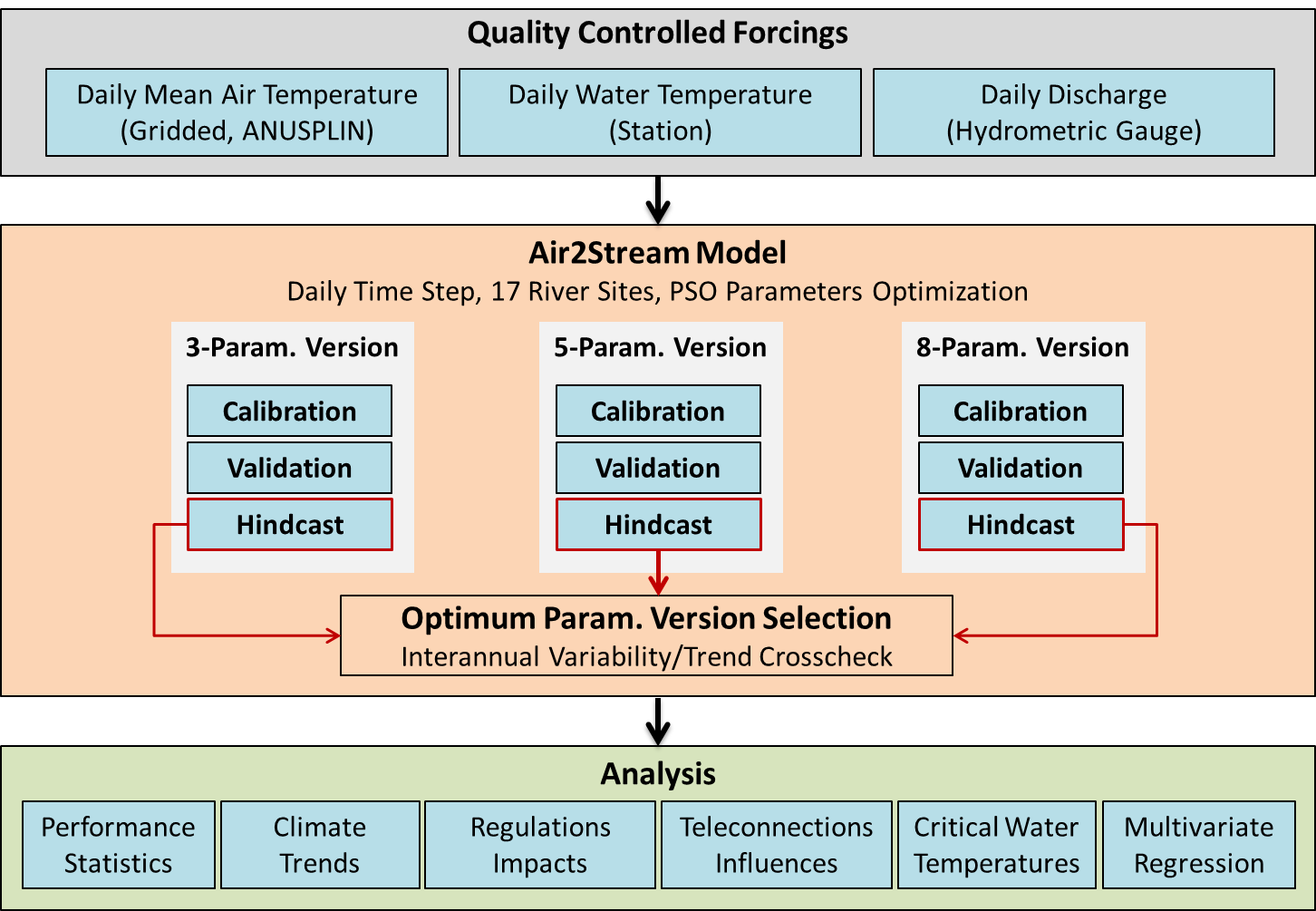** |
| --- |

**Supplementary Figure S1.** Block diagram highlighting the Air2Stream experimental design for each river site.

| 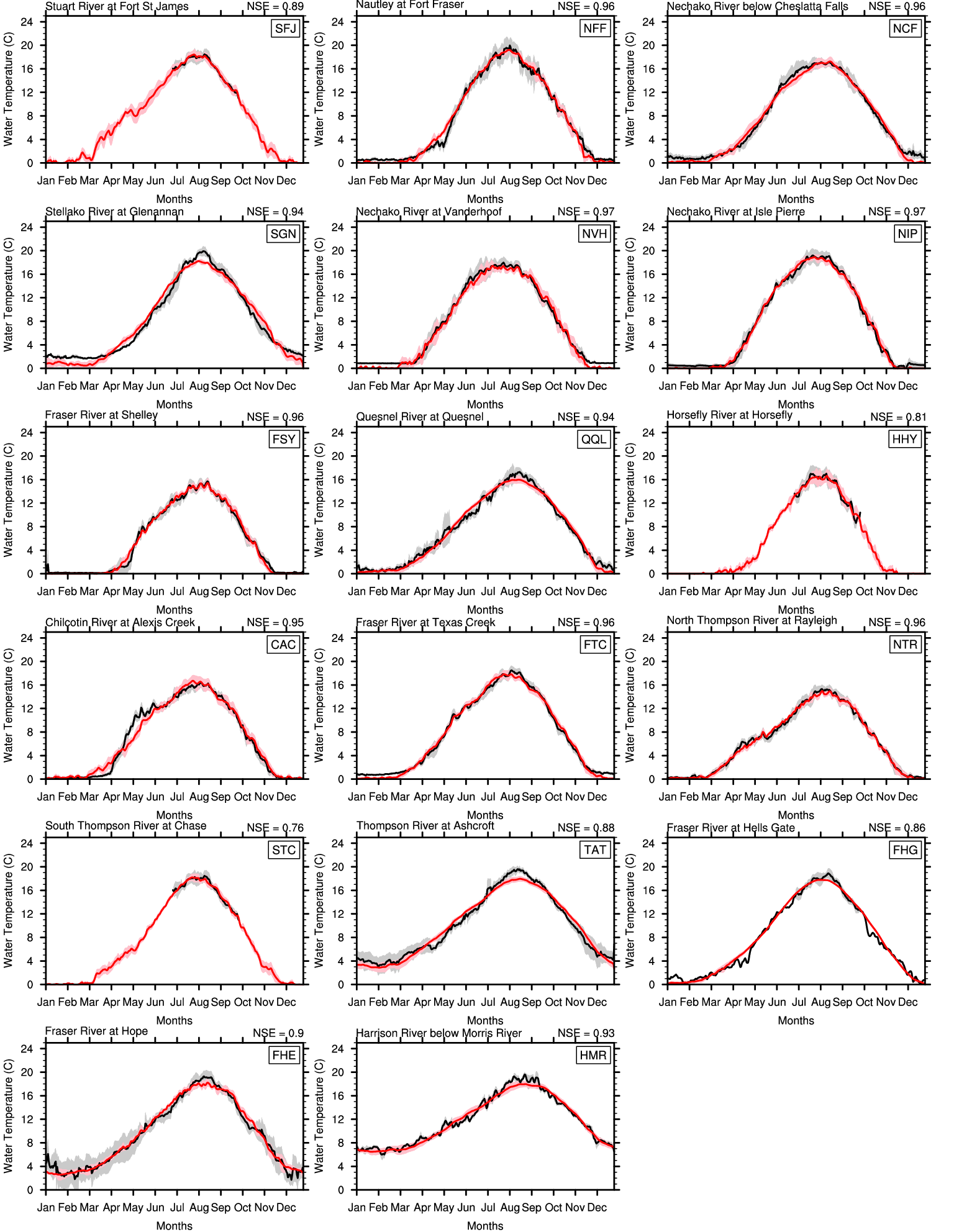 |
| --- |

**Supplementary Figure S2.** Observed (black) and simulated (pink) daily mean water temperatures for the Air2Stream model calibration periods with observed water temperature data. Shading represents daily water temperatures in individual years for 5-95% ranges. See Supplementary Table S3 for calibration time periods.

| 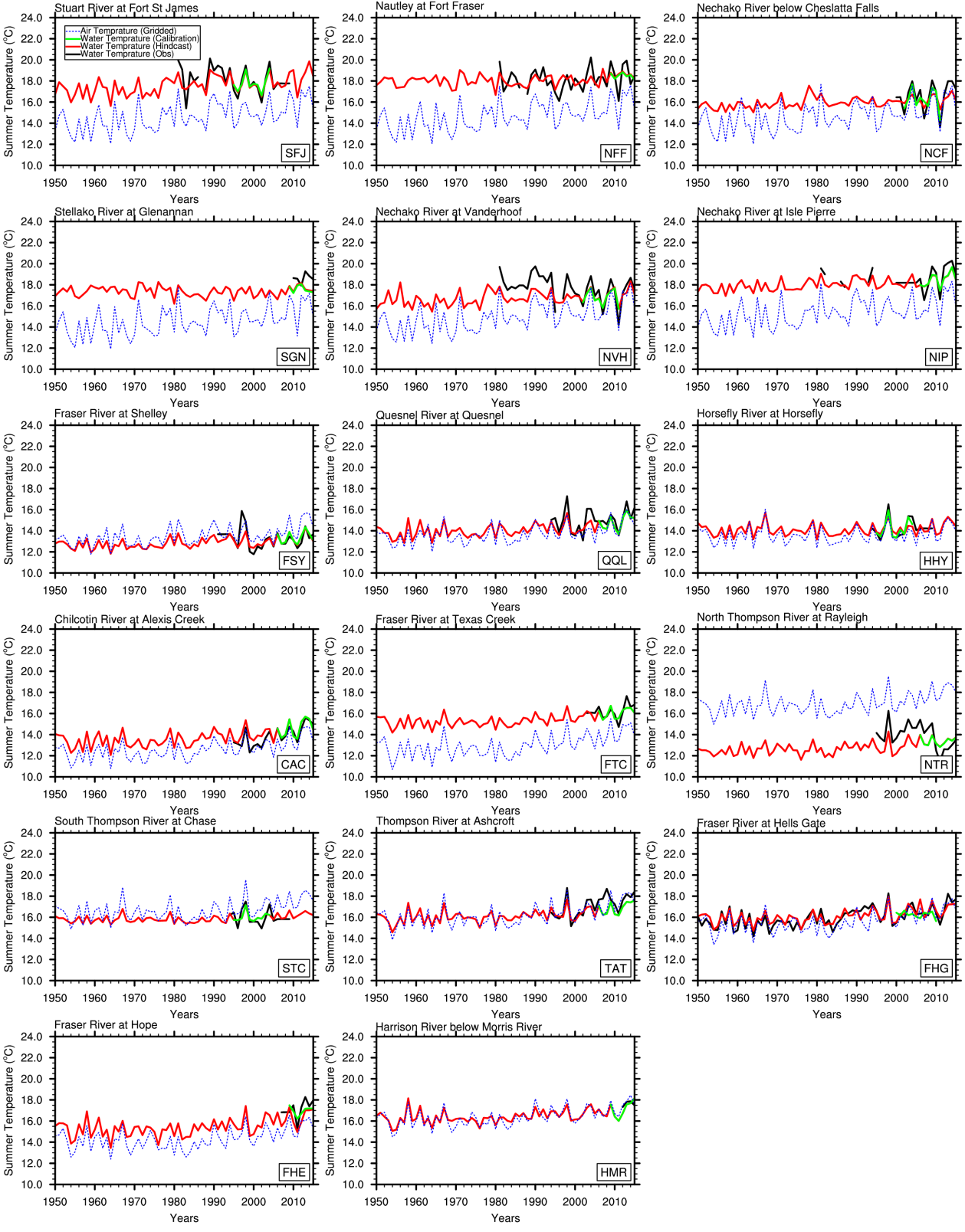 |
| --- |

**Supplementary Figure S3.** Interannual variability of observed (OBS) and simulated mean summer water temperatures for 1950-2015 (hindcast and calibration simulations). Observed and calibrated water temperature records vary for sites as per data availability (see Supplementary Table S1). Dotted blue lines depict ANUSPLIN-based gridded summer mean air temperatures. At SFJ, NFF, NCF, SGN, NVH and NIP, summer mean was calculated from 20 July to 20 August.

| **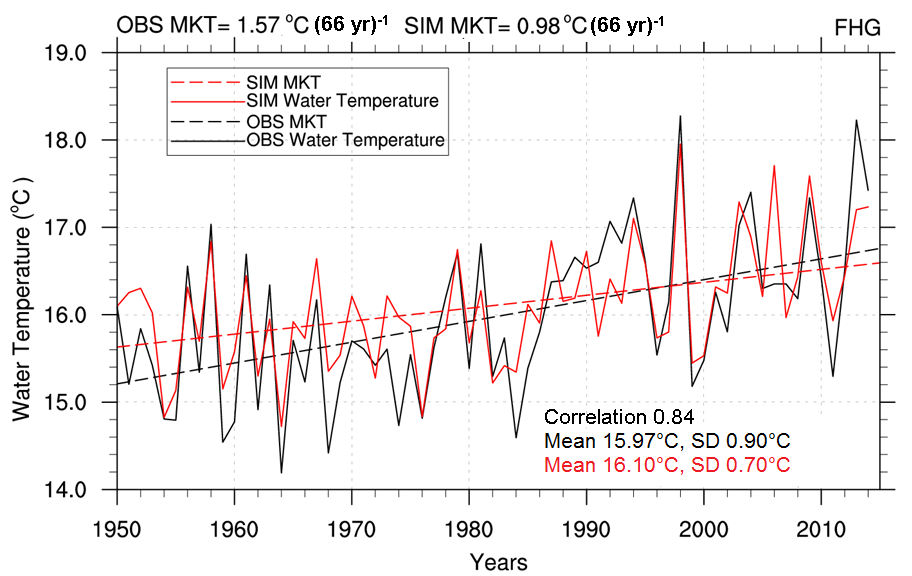** |
| --- |

**Supplementary Figure S4.** Comparison of observed and simulated mean summer water temperatures for the Fraser River at Hells Gate (FHG), 1950-2015. Dashed lines represent monotonic trends significant at *p*<0.05 and inferred from the Mann-Kendall Test (MKT).

| 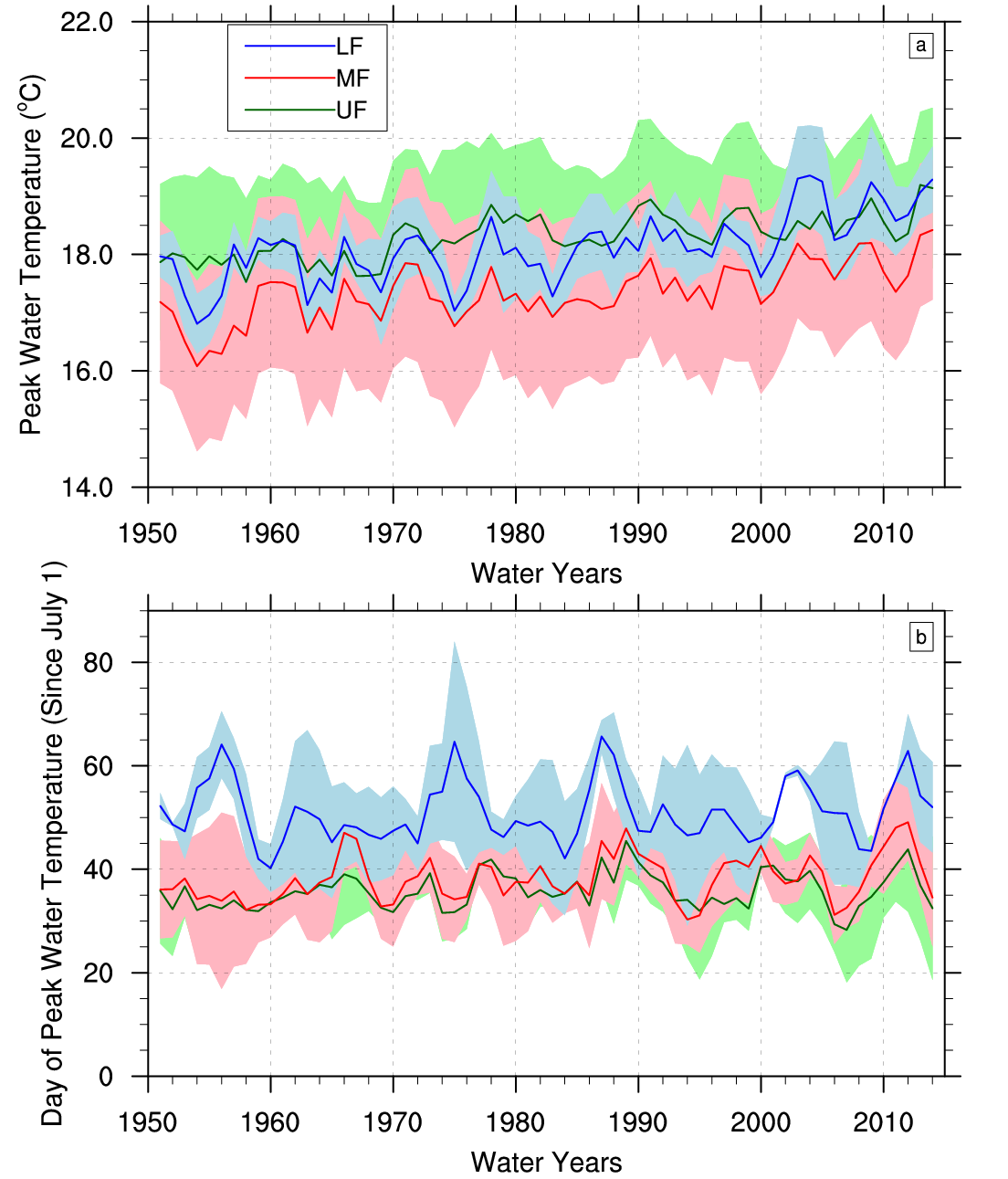 |
| --- |

**Supplementary Figure S5.** Simulated (a) maximum water temperature and (b) corresponding day in summer of the maximum water temperature across river sites split into the upper, middle and lower Fraser.

| 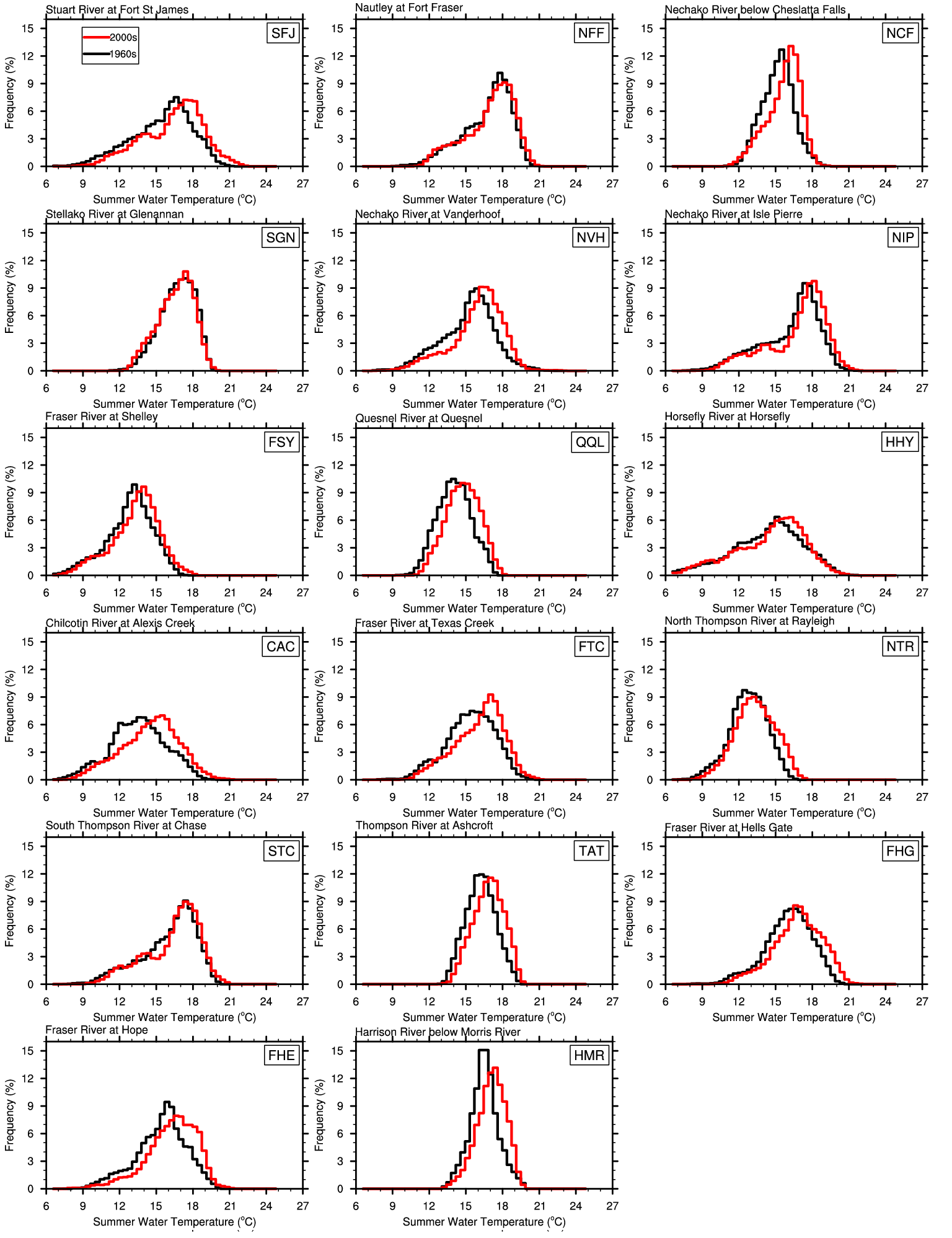 |
| --- |

**Supplementary Figure S6.** The frequency distributions of simulated mean summer water temperatures for the 1960s and 2000s. 5-point running means were applied to smooth all distribution curves to clarify the comparison between time periods. Statistical significance was computed based on the difference of distributions across years for each site. The frequency distributions do not differ significantly at *p* < 0.05 according to a Kolmogorov‐Smirnov two‐sample test.

| 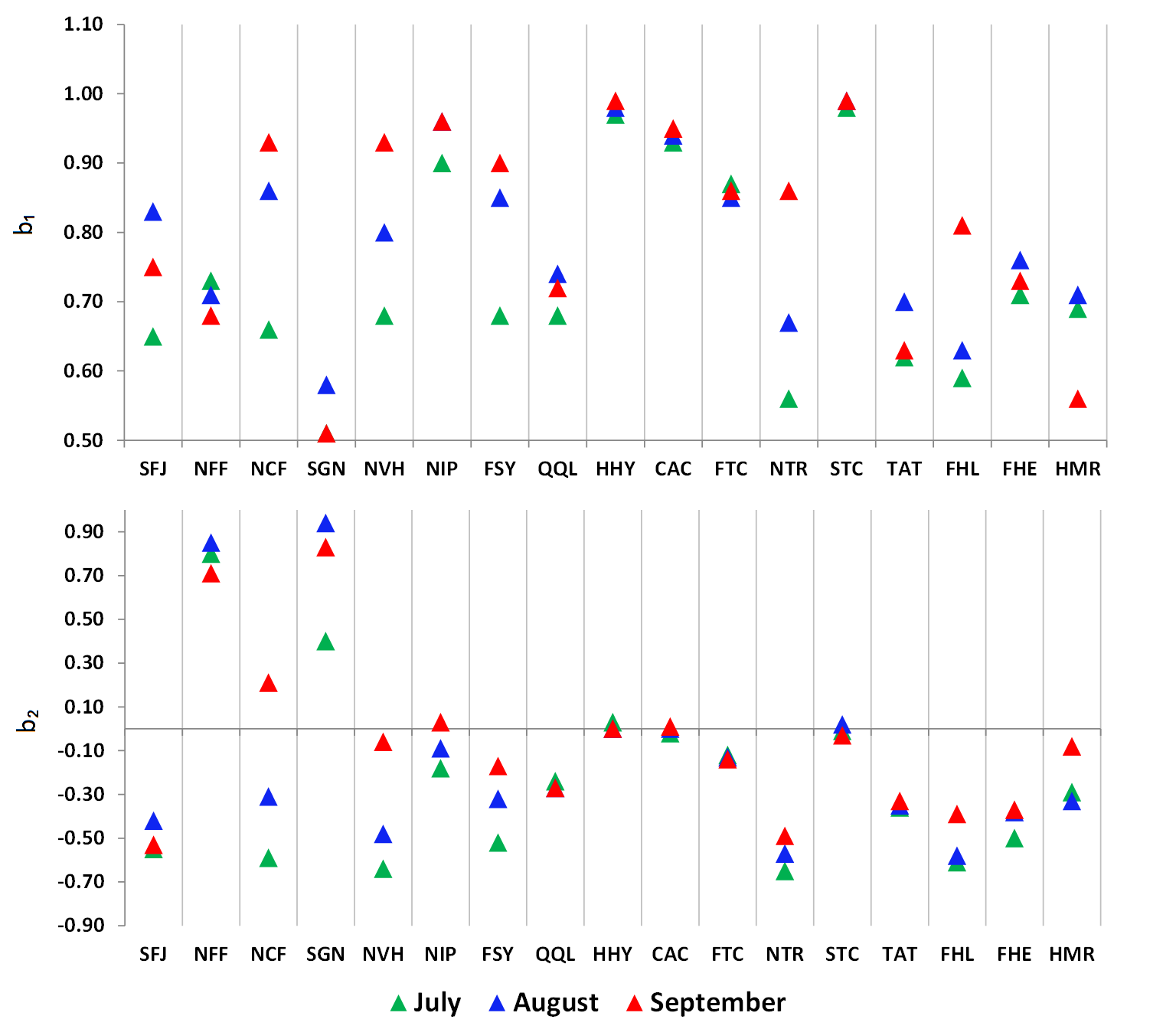 |
| --- |

**Supplementary Figure S7.** Comparison of multivariate linear regression (MLR) b_1_ and b_2_ standardized coefficients for summer months. MLR estimated annual water temperature using gridded air temperature, observed discharge and simulated water temperature data for 1950-2015.
